# Power of Proteomics in Linking Oxidative Stress and Female Infertility

**DOI:** 10.1155/2014/916212

**Published:** 2014-05-12

**Authors:** Sajal Gupta, Jana Ghulmiyyah, Rakesh Sharma, Jacques Halabi, Ashok Agarwal

**Affiliations:** Center for Reproductive Medicine, Cleveland Clinic Foundation, 10681 Carnegie Avenue, Desk X11, Cleveland, OH 44195, USA

## Abstract

Endometriosis, PCOS, and unexplained infertility are currently the most common diseases rendering large numbers of women infertile worldwide. Oxidative stress, due to its deleterious effects on proteins and nucleic acids, is postulated to be the one of the important mechanistic pathways in differential expression of proteins and in these diseases. The emerging field of proteomics has allowed identification of proteins involved in cell cycle, as antioxidants, extracellular matrix (ECM), cytoskeleton, and their linkage to oxidative stress in female infertility related diseases. The aim of this paper is to assess the association of oxidative stress and protein expression in the reproductive microenvironments such as endometrial fluid, peritoneal fluid, and follicular fluid, as well as reproductive tissues and serum. The review also highlights the literature that proposes the use of the fertility related proteins as potential biomarkers for noninvasive and early diagnosis of the aforementioned diseases rather than utilizing the more invasive methods used currently. The review will highlight the power of proteomic profiles identified in infertility related disease conditions and their linkage with underlying oxidative stress. The power of proteomics will be reviewed with regard to eliciting molecular mechanisms for early detection and management of these infertility related conditions.

## 1. Introduction

Current studies estimate that 17% of couples are confronted with an inability to conceive and their infertility could stem from different causes, either affecting the male or the female. Around 50% of these are due to the female factor and associated diseases in the female reproductive tract [1]. When defining infertility, it has been noted that couples are termed infertile if they are unable to conceive after 12 months of unprotected and regularly timed sexual intercourse [2]. The desire to procreate is naturally present in women worldwide. Due to various advances in medical techniques it has now become possible for many women to fulfill the hope of mothering children of their own. The longing to conceive a child has been recently proposed to be one of the risk factors for women's depression. Furthermore, the added financial burden is an impediment to economic status as these women are more likely to end up paying for assisted reproductive techniques (ART). Several medical advances during the past decade and a half have led to the establishment of ART clinics worldwide that provide services to infertile couples to help them achieve pregnancy. Chachamovich et al. showed that women were more likely to be psychologically distressed due to infertility than men and that they suffer from poor quality of life once they are diagnosed as infertile [3].

There seems to be multiple reasons for female infertility, of which the most common are endometriosis related infertility, ovulatory disorders, tubal factor infertility, and unexplained infertility. Women suffering from any of the above-said diseases have been shown to have a lower chance of conceiving a child and are more likely to seek infertility treatment [4].

Several studies have linked the prevalence of female infertility with an increase in oxidative stress levels in the various critical micro- or macroenvironments in the body. Oxidative stress results from an increase in reactive oxygen species (ROS), as the antioxidant capacity of the cells to scavenge and remove these free radicals decreases. ROS is the byproduct of the process of respiration taking place in the mitochondria [5]. Commonly, ROS molecules interact with proteins, lipids, carbohydrates, or DNA molecules within cells, causing damage to cellular structures including cell membranes and genetic material [6]. The imbalance of ROS can eventually lead to epigenetic differences and changes in cellular pathways and transcription factors [7]. Studies have shown that oxidative stress causes alterations in certain protein pathways and the abnormal expression of several proteins could possibly lead to the pathophysiology of female infertility. The abnormally expressed proteins could be integral factors responsible for oxidative stress reaction, or antioxidants or different binding proteins that may bind oxidants and antioxidants such as superoxide dismutase (SOD), paraoxonase (PON), hemopexin, apolipoproteins, and heat shock proteins [8–13].

The study of the expression of different proteins in a cell or tissue in a temporal and spatial fashion is referred to as proteomics [14]. This technique is fairly new and has been advancing at an extraordinary pace, as new methodologies evolve for the detection of minute amounts of proteins in different body fluids. Since proteins dictate cellular functions to a large extent, comparative proteomics investigating various proteins in the normal and diseased samples is deemed to be an important factor in the diagnosis and treatment of diseases [15]. Comparative proteomic analyses could possibly aid in the identification of biomarkers for noninvasive diagnosis of female diseases and assist in the prediction of success rates for the assisted reproduction techniques (ART).

The purpose of this review is to examine the relationship between oxidative stress and differentially expressed proteins in female infertility that has been identified by proteomic techniques. The review will focus on the proteins involved in endometriosis, polycystic ovarian syndrome, and unexplained infertility and those involved in examining the embryonic secretome associated with ART. Finally, our review will illustrate the power of proteomics in the discovery of biomarkers for noninvasive diagnosis as well as prediction of ART success rate.

## 2. Oxidative Stress and Female Fertility

Reactive oxygen species (ROS) are a class of free radicals lacking one or more electrons in their outer shell. They are highly reactive and have a tendency to interact with neighboring molecules to stabilize their structure. The electron transport chain in the mitochondria is an important endogenous source of ROS. Several exogenous factors, namely alcohol, smoking, and environmental factors, can enhance the ROS production [16]. The free radicals commonly associated with oxidative stress include hydroxyl radicals, superoxide anion, and hydrogen peroxide [17].

Enzymatic antioxidants such as glutathione oxidase and superoxide dismutase as well as nonenzymatic antioxidants (vitamin A, vitamin E, zinc, and selenium) are essential in maintaining adequate levels of ROS in the cell by disposing and removing excess free radicals [7]. Any disruption in the antioxidant/ROS balance leads to a state of oxidative stress in the cell with damaging consequences.

Physiological levels of ROS are required for proper functioning of different biological pathways and in maintaining homeostasis within the human body. Low levels of free radicals act as modulators in female reproductive pathways such as oocyte maturation, physiological follicular atresia, ovulation fertilization, luteal regression, and corpus luteum formation during pregnancy [18]. At puberty, monthly maturation of a primordial follicle into a graafian follicle occurs. Normal levels of ROS help in the resumption of meiosis-I and also in the development of a dominant oocyte, and increased concentrations of antioxidants have been shown to inhibit this process. Conversely, antioxidants promote resumption of meiosis-II [19] allowing for fertilization at the metaphase II level. Therefore, follicle maturation is a classic example of the delicate balance that exists between ROS and antioxidants in the maintenance of the regulated sequence of events that culminate in ovulation. As the follicle grows, hormonal secretions increase and result in elevation of Cytochrome P450. Cytochrome P450 enzymes can undergo changes in the absence of a substrate to produce ROS including superoxide anions, hydroxyl radicals, and hydrogen peroxide. CYP 2E1, one of the enzymes in the P450 complex, is thought to induce NADPH-dependent lipid peroxidation. Another component of the P450 complex, CYP4A, is associated with the production of superoxide and hydrogen peroxide [20]. The increase in ROS in pre-ovulatory follicle is crucial for the onset of ovulation [5].

ROS is also believed to play a role in the different phases of the endometrial cycle. Late luteal phase is characterized by elevated levels of lipid peroxide and a decrease in the antioxidant, superoxide dismutase. ROS stimulates the secretion of PG2F*α* through activation of NFk*β* (nuclear factor-kappa*β*) [21]. Decreased levels of estrogen and progesterone lead to a decreased SOD expression and hence generate oxidative stress in the uterus, resulting ultimately in endometrial shedding and lack of implantation. Controlled levels of ROS have, however, been associated with the angiogenic activity in the endometrium causing regeneration in every cycle. These studies show that limited levels of ROS are necessary to maintain physiological function, but when present in higher concentrations, ROS can have deleterious effects [22].

Disruption in physiological levels of ROS leads to female reproductive dysfunction and in some cases, to unexplained infertility. Oxidative stress in female reproduction has been associated with polycystic ovarian syndrome and endometriosis. Needless to say, these pathologies negatively affect pregnancy rates and IVF outcomes.

ROS is produced at multiple sites in a preovulatory follicle and renders it, a susceptible medium for oxidative stress. ROS levels can be measured in several different media including the follicular fluid (FF), endometrial fluid, and peritoneal fluid. The FF contains ROS producing agents, including cytokines, neutrophils, and macrophages. Steroidogenesis involves a series of enzymatic reactions which result in the formation of steroid hormones. The monooxygenase pathway is mediated by p450 and results in production of ROS [23] rendering the FF as a suitable medium for oxidative stress measurement.

Even though abnormal increase in ROS levels in women is negatively correlated with (1) oocyte development [24], (2) embryonic development [25], and (3) pregnancy outcome [26], physiological levels of ROS are required for healthy development of oocytes and better IVF outcomes [27]. This makes it important to determine the threshold value of ROS, beyond which its negative effects in fertility are observed. Jana et al. determined the upper cut-off value of ~100–107 counted photons per sec (cps) of ROS in follicular fluid, above which ROS negatively affects fertility and IVF results. In women with tubal factor infertility, undergoing IVF, ROS cut-off levels were determined to be 100 counted photons per second (cps). With ROS levels lower than 100 cps, embryo formation and embryo quality were enhanced compared to those with ROS levels above 100 cps [28]. When the same study was performed on patients with PCOS and endometriosis, the upper cut-off value was identified to be 107 cps. The authors conclude that women with ROS levels higher than 107 cps may encounter difficulties conceiving a viable embryo [28]. The result of this study stresses the importance of antioxidants to suppress ROS buildup and maintain physiological levels of free radicals for proper cell functioning and homeostasis.

### 2.1. Unfavorable Balance of ROS versus Antioxidants in Endometriosis

Researchers have examined that high levels of ROS can impact fertility in patients with endometriosis. Szczepańska et al. compared the antioxidant levels of superoxide dismutase in the peritoneal fluid in infertile women with (1) endometriosis, (2) unexplained infertility, and (3) fertile women. The lowest concentrations of superoxide dismutase were found in the first group of patients [29]. Similar findings were reported by Liu et al. who demonstrated decreased amounts of superoxide dismutase in the peritoneal fluid of infertile women with endometriosis when compared with fertile women [30]. Therefore, an imbalance between ROS levels and antioxidants can explain infertility in patients with endometriosis. In a study by Prieto et al., the concentration of vitamin C was found to decrease in the follicular fluid of patients with endometriosis compared to infertile patients without endometriosis. This could be due to the excessive consumption of the antioxidant in order to neutralize the abnormal levels of ROS [31]. Furthermore, decreased levels of vitamin C have been associated with ovarian atrophy and follicular atresia implicating its importance in female infertility [32]. Although ROS levels were determined to be crucial in promoting infertility in patients with endometriosis, there is little agreement on oxidative stress markers, due to the inconsistency in results across different studies [31]. According to Jackson et al., higher concentration of Vitamin E was reported in the follicular fluid of patients with endometriosis [33]. Conversely, concentration of vitamin E was lower in the peritoneal fluid of patients with endometriosis [34]. This discordance in results may be explained by the vitamin E rich diet or the nutrient supplementation that is commonly suggested for women suffering from endometriosis [31]. It may also be due to the increased consumption of vitamin E in the peritoneal fluid window.

Retrograde menstruation is another theory for endometriosis and seems to also lead to increased oxidative stress through a different mechanism. It may also be observed in women with normal menstruation and can subsequently cause an increase in iron levels in the female pelvis. The increased iron is taken up by macrophages, therefore reducing the ability of ferritin to remove this heavy metal. The lack of antioxidant activity of ferritin therefore leads to oxidative stress. The increase in oxidative stress causes change in the cellular function due to changes in the genetic and protein content and consequently, the release of inflammatory molecules [10]. Endometriosis has been reported to be associated with worsening of IVF outcomes, due to impaired oocyte quality, decreased fertility, and compromised implantation rates. The increase in ROS in endometriosis patients can lead to adverse effects on embryo such as intrauterine growth restriction, abortions, or fetal dysmorphogenesis [35]. DNA damage was the highest in granulosa cells from patients with endometriosis which resulted in fewer good quality embryos in patients undergoing IVF [36].

### 2.2. Increased ROS Levels in Polycystic Ovarian Syndrome

Oxidative stress has been implicated in different female diseases including polycystic ovarian syndrome (PCOS). Various studies reflect the presence of oxidative stress in PCOS patients. In a study by Hilali et al., PCOS patients had increased serum prolidase activity as well as total oxidant status and oxidative stress index, which is the ratio of oxidants to total antioxidants status. Prolidase is a matrix metalloproteinase (MMP) that degrades the extracellular matrix. It was found that increased MMPs can result in symptoms seen in women with PCOS such as abnormal ovarian extracellular remodeling, multiple cyst formation, and chronic anovulation, leading to infertility [37].

Furthermore, infertility in PCOS patients was related to the effect of follicular fluid's oxidative stress levels on the meiotic spindle formation in the oocyte. Fertilization rates and embryo quality were found to be decreased in these women, when compared to women suffering from tubal factor disease and accompanied by increased levels of ROS. These data and other studies indicate that elevated ROS in the follicular fluid tends to limit the fertilization potential of oocytes, through disruption of meiotic spindle formation [38].

### 2.3. Association of Increased ROS Levels in Unexplained Infertility

By definition, unexplained infertility is considered as a “diagnosis of exclusion” when no known factors are responsible for infertility [39]. Studies utilizing malondialdehyde (MDA), a marker of lipid peroxidation [40], have implicated the role of oxidative stress in patients with unexplained infertility. Conversely, it was also seen that glutathione was decreased in the peritoneal fluid of patients with idiopathic infertility [41]. Hence, one theory is that underlying cause of unexplained infertility is the disruption of the delicate balance that exists between ROS and antioxidant levels [41].

Selenium is part of the selenoenzyme glutathione peroxidase and protects the integrity of membrane structures by enzymatically degrading endogenous and exogenous hydrogen peroxide and lipid peroxides. Selenium levels in follicular fluid of infertile patients who presented for IVF treatment were measured [42]. The women were divided into three groups depending on the etiology of infertility: tubal factor, unexplained, and male factor. Selenium and glutathione peroxidase levels were found to be the lowest in patients with unexplained infertility. Consequently reduced amount of antioxidants in ovarian follicle could be a reason for unexplained infertility [42].

## 3. Power of Proteomics in Studies of Female Infertility

Post-translational modifications occurring within cells are mainly responsible for the discrepancies noted between the genome and the expressed proteome. Changes that occur at the gene level are not always representative of the processes taking place inside the cell, as the proteins exert their multitudes of functions via more complex and diverse structures, generated through posttranslational modifications and protein-protein interactions [13]. Currently, ~300 different types of PTM are responsible for the huge repertoire of proteins originating from a small number of genes [43]. This leads to diversity in the function and number of proteins, far more complex than visualized by the number of genes identified by the human genome project. It is, however, the proteins that finally dictate the function of each cell through dynamic interactions with their environment and with each other [15]. Attention has now shifted to the protein complement of the genome, as the one gene-one protein concept that had served as a landmark in molecular biology is no longer true.

With the advancement in technology for studying proteins, it has become possible to focus on the role of proteomics in different fields of medicine. The word “omics” refers to the study of a certain field, in this case the proteins under investigation. This includes study of the protein content of a cell or a tissue, providing insight and ideas for biomedical interventions and identification of potential biomarkers [14]. Proteins differ greatly between two cells, between the same cell at different times and between normal and abnormal cells. Complexity in protein function has driven investigators to probe the protein factors responsible for both male and female infertilities by studying tissues and bodily fluids of the reproductive tracts as well as in pathological tissues. Also, proteomic analysis could aid in the identification of potential “fertility proteins” that could prove highly beneficial in the diagnosis of infertility. In this era of proteomics, it is important that proteomic experiments are reproducible from one lab to the other. Standard procedures for sample collection, handling, storage, and analysis need to be adopted, as they will be important for identification of biomarkers in disease development and progression [44].

Advanced research has led to a better understanding of the underlying causes and the mechanistic pathways of female infertility [45]. Numerous studies have been conducted on endometrial tissue and secretions, endometrial receptivity, ovaries, uterine fluid, follicular fluid, peritoneal fluid, plasma, endometriotic tissue, and tissues from PCOS patients [8, 13, 31, 46–48]. However, the collection of reproductive samples is a challenge as some of the samples have to be obtained invasively [44]. The bigger challenge is the analysis of the diverse microenvironments since they not only contain proteins of interest, but also growth factors, inflammatory factors, different cells, and several biological molecules. This is a limitation in proteomics as the samples have very high concentration of plasma proteins such as albumin that should be removed in order to determine the protein content of plasma related to endometriosis [47]. Another limitation is the retrieval of adequate amount of the sample and hence the need for techniques with high specificity and sensitivity to measure small amounts of proteins [48].

The ability of proteomics to characterize certain body fluids and determine their contents makes it a powerful tool in today's world [48]. It can be used for the determination of biomarkers specific to certain diseases as well as to differentiate between fertile and infertile women. The search for certain proteins that can discriminate a receptive endometrium from one that is less receptive continues, as it has great potential in increasing the success rates associated with natural fertility as well as in assisted reproductive techniques. Considerable progress has been made in classifying embryonic proteins, which will aid single embryo selection during assisted reproductive techniques, thereby making the process of ART more efficient and decreasing unwanted multiple gestation rates as well as failed procedures [4].

## 4. Methodology of Proteomic Analyses in Infertile Females

The separation and identification of proteins are currently performed using different methods depending on the requirements of each experiment. Techniques are continuously modified to achieve more accurate and specific results. Physical factors such as molecular mass, acidity, charge, and amino acid content of proteins determine the design of the experiment.

The most commonly used procedures are a systematic two-dimensional gel electrophoresis (2-DE) for separating the proteins, mass spectrometry for identifying them, and Western blotting for confirmation. Integral to the purity of samples, it is essential that before separation, samples should be denatured, purified, and solubilized for complete disintegration of internal bonds resulting in single proteins [49].

### 4.1. Two-Dimensional Gel Electrophoresis (2DE) and Difference Gel Electrophoresis (DIGE)

2DE is an integral tool in the study of proteomics. It is a classical method often described as the proteomic “workhorse.” It enables the separation of complex protein mixtures by combining isoelectric focusing (IEF) in the first dimension and SDS PAGE in the second dimension [50]. Protein profiles can be visualized using both fluorescent and visible stains. It is usually used to find biomarkers in various disease states. 2DE has poor reproducibility and limited sensitivity.

Difference gel electrophoresis (DIGE), in which two protein samples are separately labeled with different fluorescent dyes and then coelectrophoresed on the same 2DE gel, was developed to overcome the reproducibility and sensitivity limitations. DIGE utilizes mass- and charge-matched, spectrally resolvable fluorescent dyes (Cy2, Cy3, and Cy5) to label up to 3 different protein samples (or 2 samples and 1 pooled internal standard)* in vitro* prior to 2D electrophoresis [51]. The fluorescent dyes have been designed so that they do not alter the charge of proteins or impart significant electrophoretic mobility differences to identical proteins. Both the control and the experimental samples are run in a single polyacrylamide gel but imaged separately (Typhoon 9410) at a discrete wavelength for each respective dye. Hence, the images are overlaid without any “warping” which substantially raises the confidence with which protein changes between samples can be detected and quantified.

Quantitative and comparative proteomics can be done using 2D-DIGE (two-dimensional difference gel electrophoresis). An automated software program is used to detect, quantify, and annotate differentially expressed proteins. 2D-DIGE offers all the advantages of 2D-PAGE and overcomes the inherent disadvantage of variation and reproducibility problem in a 2D-PAGE. These procedures are powerful technologies for comparing complex protein mixtures from biological samples in proteomics research. Compared to traditional 2-D gel techniques for protein profiling, DIGE provides a significantly more efficient, sensitive, and reliable way to detect proteins whose expression is altered between control and treated samples.

There are many advantages to 2D-DIGE over traditional 2D gels: (1) high sensitivity: CyeDye fluorescent dye has a sensitivity of 0.2 ng/spot versus 100 ng/spot with the traditional 2D gels stained by Coomasie; (2) high accuracy: extremely high spot resolution allows accurate software-aided spot quantitation and protein expression comparison between samples. Differences as small as 10% can be detected and protein isoforms and posttranslational modifications can be easily visualized by 2D-DIGE. While DIGE provides a reliable and sensitive platform for discovering proteome changes in an unlimited variety of circumstances, conventional 2DE is still needed to identify the proteins [52, 53].

### 4.2. Mass Spectrometry

The principle of mass spectrometry is applicable to all molecules including lipids and proteins. In proteomics principle of mass spectrometry depends on the separation of the peptides according to their mass-to-charge ratio. An ionizer bombards the proteins with hydrogen ions and results in a charge on the protein as they pass through the mass analyzer to the detector which is attached to a computer program [54].

For conducting gel-based proteomics studies, the region of gel containing the protein of interest is excised, and the sample is digested “in-gel” with trypsin (which cleaves proteins on the C-terminal side of either lysine or arginine), the proteins are then eluted from acrylamide. These peptides are then subjected to MALDI-TOF, producing a series of peaks, each describing the molecular mass of a single peptide in the mixture.

Proteomic application of mass spectrometry (MS) is constantly growing as more improved instruments and smarter ways to couple them become available. The most commonly used mass analyzers for protein biochemistry applications are time-of-flight (TOF), triple-quadrupole, quadrupole-TOF, ion trap instruments, and hybrid ion-trap orbitrap instrument.

Four common uses of MS based proteomics are (1) protein identification, (2) protein sequencing, (3) identification of posttranslational modifications, and (4) characterization of multiprotein complexes. There are other complex MS analyzers such as the Fourier transform-ion cyclotron resonance MS (FT-ICR). There are also other MS applications, such as structural analysis. The principle of mass spectrometry is applicable to all molecules including lipids and proteins.

### 4.3. Matrix Assisted Laser Ionization/Desorption Mass Spectrometry

In the tissues from patients with endometriosis, protein bands are excised and subjected to further identification using one of the most common ionization techniques used in biology which are matrix-assisted laser desorption ionization (MALDI) and electrospray ionization (ESI). MALDI is most commonly used to produce a preliminary scan of the peptide components released from a sample by proteolytic digestion, such as the proteins present in an acrylamide gel slice. It is a solid phase technique in that researchers mix a peptide sample with a huge excess of matrix material, usually either a-cyano-4-hydroxycinnamic acid or dihydrobenzoic acid, and precipitate the mixture on a plate by drying. Whereas MALDI is a solid-state and pulsed process, ESI is a liquid phase and is usually continuous technology. ESI is compatible with both high-pressure liquid chromatography (HPLC) and capillary electrophoresis, both of which may be used to concentrate and purify individual peptides prior to mass analysis. MS instruments, despite their names, do not actually provide a mass value. Instead, they report the mass-to-charge (*m*/*z*) ratio. If the charge of the ion is known, the mass can be calculated. The most commonly used mass analyzers for protein biochemistry applications are time-of-flight (TOF), triple-quadrupole, quadrupole-TOF, ion trap instruments, and hybrid ion trap Orbitrap instruments. The TOF analyzer is conceptually the simplest spectrometer [54]. Typical configurations for biological applications are MALDI-TOF and ESI coupled to an ion trap, triple-quad, Q-TOF, or orbitrap.

### 4.4. Surface-Enhanced Laser Desorption/Ionization

Surface-enhanced laser desorption/ionization (SELDI) is an ionization method in mass spectrometry that is used for the analysis of protein mixtures. SELDI is typically used with time-of-flight mass spectrometers and is used to detect proteins in tissue samples, blood, urine, or other clinical samples. Comparison of protein levels between patients with and without a disease can be used for identification of new biomarkers. SELDI-TOF MS is a novel approach to biomarker discovery that combines two powerful technologies: chromatography and mass spectrometry. SELDI-TOF-MS is a variation of matrix-assisted laser desorption/ionization (MALDI) that uses a target modified to achieve biochemical affinity with the analyte compound.

In SELDI, the protein mixture is spotted on a surface modified with a chemical functionality. Some proteins in the sample bind to the surface, while the others are removed by washing. After washing the spotted sample, the matrix is applied to the surface and allowed to crystallize with the sample peptides. Binding to the SELDI surface acts as a separation step and the subset of proteins that bind to the surface are easier to analyze. SELDI technology was commercialized in 1997 as the Protein Chip system and is marketed by Bio-Rad Laboratories. The Protein Chip platform consists of chips presenting specific chromatographic surfaces, including reverse phase, anionic exchange, cationic exchange, and immobilized metal affinity surface.

SELDI has been applied in identifying diagnostic markers in ovarian, prostate, breast, bladder, hepatic, and pancreatic cancer using serum or plasma. The technique has also been used to characterize phosphorylated and glycosylated proteins, transcription factors, and peptides and proteins shed or secreted by various cancer cell lines. SELDI-TOF-MS is a technology that can produce proteomic “fingerprints” from biological samples using a relatively high throughput platform. Posttranslationally modified proteins can also be detected using SELDI-TOF MS.

An application that has generated much interest in SELDI-TOF MS is its promise as a diagnostic tool in the early detection of diseases, such as ovarian cancer. Serum samples taken from healthy and diseased subjects are spotted onto protein chips and analyzed by TOF MS to generate a protein profile.

SELDI-TOF MS has its limitations. The system is manual, time consuming, and prone to human error. The low resolution, and hence mass accuracy, coupled with the inability to do TOF MS/MS, prevent reliable identification based on conventional bioinformatic searching. Although a SELDI interface is available for higher-resolution instruments, such as the hybrid quadrupole TOF instrument that has a much higher mass accuracy and MS/MS capabilities, the routine application of these instrument platforms for biomarker identification directly from the applied sample has yet to be demonstrated. Largely successful at discovering proteins in the low-molecular-weight range, SELDI-TOF MS has not yet shown itself consistently successful in studying high-molecular-weight proteins.

SELDI-TOF MS provides a simple, low-resolution pattern generated from proteins retained on a specific chromatographic surface. In most instances, it does not allow the direct identification of proteins that may be potential disease biomarkers. SELDI-TOF MS can rapidly screen and generate proteomic patterns for hundreds of crude samples. This simple answer may have a profound impact on how proteomics is viewed in the future.

Bias in serum specimens of early studies, differences in study design, and limitations of proteins detected by SELDI-TOF MS in unfractionated serum may explain the inability of this study to identify patients especially in studies on patients with colorectal cancer.

There are many methods of mass spectrometry-based proteomics that do not use TOF. For example, the Q-Exactive (Thermo) utilizes a quadrupole-orbitrap hybrid mass spectrometer and is the most widely used platform for shotgun proteomics. Waters' MSE platform is based on a very good TOF analyzer but depends on a Quadrupole analyzer (hybrid) in line. Moreover, the introduction of ion mobility is one of the backbones of their technology. The newer Triple TOF (AB Sciex) is in essence a quadrupole-time of flight hybrid as well. Even FT-ICR MS has been used due to its very high resolution.

### 4.5. Liquid Chromatography-Mass Spectrometry

Liquid chromatography-mass spectrometry (LC-MS) is a hyphenated technique, which combines the separation power of LC with the detection power of mass spectrometry. Liquid chromatography-mass spectrometry (LC-MS, or alternatively HPLC-MS) is an analytical chemistry technique that combines the physical separation capabilities of liquid chromatography (or HPLC) with the mass analysis capabilities of mass spectrometry (MS). The LC-MS system consists of four main components: (1) a chromatographic column, (2) an ionization source, (3) mass analyzer, and (4) detector. LC-MS is a powerful technique that has very high sensitivity and selectivity and so is useful in many applications.

Liquid chromatography/mass spectrometry (LC/MS) has become a powerful technology in proteomics studies in drug discovery, including target protein characterization and discovery of biomarkers. Its application is oriented towards the separation, general detection, and potential identification of chemicals of particular masses in the presence of other chemicals (i.e., in complex mixtures), for example, natural products from natural-products extracts and pure substances from mixtures of chemical intermediates.

LC-MS is also used in proteomics where again components of a complex mixture must be detected and identified in some manner. The bottom-up proteomics LC-MS approach generally involves in-solution protease digestion and denaturation. The denaturation of the tertiary structure is done using urea, trypsin enzyme is used for the degradation and the remaining cysteine residues are capped with iodoacetamide. They are then subjected to LC-MS/MS (tandem MS) to derive their individual sequences. LC-MS/MS is the most commonly used for proteomic analysis of complex samples where peptide masses may overlap even with a high-resolution mass spectrometer. There are two general approaches to LC-MS/MS analysis of complex peptide mixtures. The first is data-dependent experiments that involve MS scans followed by MS/MS analysis on the most abundant peptide ions [55]. These experiments are utilized widely in proteomics and involve the MS/MS analysis of only one peptide ion at a time.

For complex mixtures, a data-dependent analysis may not be able to sequence all of the peptides present in the sample which has led to the development of data-independent acquisition, LC-MSE. These experiments involve the MS/MS analysis of wide mass ranges resulting in the fragmentation of multiple precursors in a single scan. These experiments have been shown to provide more comprehensive protein identification [56]. Samples of complex biological fluids like human serum may be run in a modern LC-MS/MS system and result in over 1000 proteins being identified, provided that the sample was first separated on an SDS-PAGE gel or multidimensional HPLC (i.e., SCX fractionation prior to LC-MS/MS analysis).

Within a little more than a decade LC-MS/MS has transformed from an esoteric gold standard technology to an affordable, flexible, and accessible technique for most clinical laboratories. LC-MS/MS has helped in developing routine methods of high sensitivity, high specificity, high throughput, and high cost effectiveness in biochemical genetics/newborn screening, drug and toxicology testing,and endocrine testing of steroids and biogenic amines. These novel methods have made a positive impact on patient care on both economic and quality fronts. LC-MS/MS has been applied to known, established peptides and proteins biomarkers that is, targeted proteomics.

Several key limitations of LC-MS/MS have become apparent with the exponential increase of its use in clinical laboratories. They center on the following, interacting aspects of clinical LC-MS/MS: highly manual workflows, complexity of operation and maintenance of instrumentation, sample throughput limits, insufficient detection sensitivity for some analytes, and problems with detection specificity.

LC/MS plays important roles in protein structural identification and quantitative measurements in proteomics research, an integral part of the drug discovery process. Its broad applications include target protein characterization and biomarker discovery in addressing challenging issues related to drug efficacy and safety in drug discovery environment. New technological advances in this area can provide additional capabilities in using LC/MS for proteomics studies. The ability to identify proteins increases 10-fold (when comparing gel-free to gel) at least, and quantification is much more precise.

Another emerging field in proteomics research is top-down proteomics. In contrast to bottom-up experiments, intact proteins are analyzed directly using high-resolution MS and dissociated subsequently by a tandem mass spectrometer. Dissociation techniques used in top-down proteomics include collision induced dissociation (CID), electron-capture dissociation (ECD), and electron-transfer dissociation (ETD). The use of LC/MS in a top-down approach has been reported in the literature, demonstrating feasibility of this method in obtaining data on a chromatographic time scale for the first time.

Orbitrap-based mass spectrometers became a workhorse in proteomics and are also widely used in all major applications of life science mass spectrometry such as metabolism, metabolomics, environmental, food, and safety analysis. Orbitrap LC-MS technology is the recognized standard for accurate mass and high-resolution measurement. Orbitrap LC-MS technology routinely delivers the highest-resolution and mass accuracy necessary to reduce analysis times and increase confidence in results. Combined with superior dynamic range and unsurpassed sensitivity, Orbitrap platforms are the only technology available that is capable of providing all four benefits at the same time, without compromise.

### 4.6. Protein Identification and Database Searching

Once the molecular masses of the unknown proteins are established experimentally, they are compared to predetermined molecular masses of previously sequenced proteins from the reference databases. Peptide mass fingerprinting (PMF) (also known as protein fingerprinting) is an analytical technique for protein identification. The unknown protein of interest is first cleaved into smaller peptides, whose absolute masses can be accurately measured with a mass spectrometer such as MALDI-TOF or ESI-TOF. These masses are then compared to either a database containing known protein sequences or even the genome. The peptide masses are compared to protein databases such as Swissprot, which contain protein sequence information. Software performs* in silico* digests on proteins in the database with the same enzyme (e.g., trypsin) used in the chemical cleavage reaction. The mass of these peptide fragments is then calculated and compared to the peak list of measured peptide masses. The results are statistically analyzed and possible matches are returned in a results table.

Alternative methods involve the LC-MS/MS analysis of digested proteins. These experiments are carried out in a data-dependent manner which involves initial MS analysis to determine peptide molecular weight which is followed by MS/MS analysis of each peptide to determine the peptide sequence. The LC-MS/MS data is searched against a protein database which has been subjected to a theoretical digestion and the theoretical MS/MS spectra are generated for each peptide. Search programs such as Mascot, Sequest, X! Tandem, and Andromeda can then compare the experimentally observed MS/MS spectrum to the predicted peptide spectra and determine which peptides, and therefore which proteins, are the best matches. Coupling LC-MS/MS with database searches can result in 1000's of protein identifications from a single experiment [57].

There are a variety of databases available: UniProt is a comprehensive, high-quality, and freely accessible database of protein sequence and functional information, with many entries being derived from genome sequencing projects. It contains a large amount of information about the biological function of proteins derived from the research literature. The UniProt consortium comprises the European Bioinformatics Institute (EBI), the Swiss Institute of Bioinformatics (SIB), and the Protein Information Resource (PIR). UniProt provides four core databases:

1. UniProtKB (with subparts Swiss-Prot and TrEMBL), UniParc, UniRef, and UniMes;
2. UniProt Knowledgebase (UniProtKB) is a protein database partially curated by experts, consisting of two sections: UniProtKB/Swiss-Prot (containing reviewed, manually annotated entries) and UniProtKB/TrEMBL (containing unreviewed, automatically annotated entries);
3. UniProtKB/Swiss-Prot is a manually annotated, nonredundant protein sequence database. It combines information extracted from scientific literature and biocurator-evaluated computational analysis. The aim of UniProtKB/Swiss-Prot is to provide all known relevant information about a particular protein;
4. UniProtKB/TrEMBL contains high-quality computationally analyzed records, which are enriched with automatic annotation.

UniProt Archive (UniParc) is a comprehensive and nonredundant database, which contains all the protein sequences from the main, publicly available protein sequence databases.

In addition there are other databases such as the UniProt Reference Clusters (UniRef) that consist of three databases of clustered sets of protein sequences from UniProtKB and selected UniParc records. The UniProt Metagenomic and Environmental Sequences (UniMES) database is a repository specifically developed for metagenomic and environmental data.

UniParc contains protein sequences from the following publicly available databases: UniProtKB/Swiss-Prot, UniProtKB/Swiss-Prot protein isoforms, UniProtKB/TrEMBL; Vertebrate and Genome Annotation Database (VEGA), and WormBase.

Publicly available (Gene Ontology (GO) annotations from GO Term Finder and GO Term Mapper), UNIPROT, STRAP, and proprietary software packages including Ingenuity Pathway Analysis (IPA, Ingenuity Systems) and Metacore (GeneGo Inc.) as well as the STRING database and Cytoscape are available to identify the differentially affected processes, pathways, cellular distribution, and protein-protein interactions amongst proteins in the control and experimental groups, as well as aid in data integration [58–62].

### 4.7. Validation of Identified Proteins

Western blot or the protein immunoblot is a widely used analytical technique used to detect specific proteins in a sample of tissue homogenate or extract. Other related techniques include dot blot analysis, Zestern analysis, and immunohistochemistry where antibodies are used to detect proteins in tissues and cells by immunostaining and enzyme-linked immunosorbent assay (ELISA).

Biomarker validation experiments can also be performed using multiple reactions monitoring (MRM) peptide quantitation on an LC-MS/MS platform [60]. These experiments differ from those described above in that peptides from the proteins of interest are analyzed by MS/MS. Typically, three peptides are selected for each protein; up to 50 proteins can be analyzed in a single analysis, which allows proteins quantitation to be performed on multiple samples in a reasonable time frame. In addition, MRM analysis coupled with isotope dilution mass spectrometry allows for the absolute quantitation of protein abundances.

## 5. Proteomic Profiles of Females with Endometriosis

The presence of endometrial glands and stroma outside the uterine cavity is referred to as endometriosis [63]. This disease is benign and its development is primarily estrogen-dependent [47]. The manifested symptoms of endometriosis are most commonly pelvic pain and infertility as the lesions develop and establish within the peritoneal region. Although there is a delay in the diagnosis of endometriosis, it has been noted that the most common symptoms are dysmenorrhea, pelvic pain, dyspareunia, and infertility [48]. A diagnosis of endometriosis is established in 17% of women who present with primary infertility, 5–21% of women with pelvic pain, and 50% of adolescents with dysmenorrhea [64]. These statistics are evidence of the prevalence of this disease among women of a wide age range, making endometriosis one of the most investigated gynecological disorders.

The prevalence of endometriosis is the highest among women of reproductive age group and declines with age, making it a topic of interest to researchers [65]. It was noted that an early diagnosis of the disease greatly reduces the risks and symptoms associated with the disease. However, this has proven to be more challenging than initially thought since the only method of diagnosis of endometriosis is by invasive techniques such as laparoscopy [47]. General fear of medical intervention prevents women from undergoing the procedure unless they are symptomatic or have already been diagnosed. According to Husby et al., the time of delay between pelvic pain and final diagnosis of endometriosis was a mean of 6.7 years [66]. However, since studies have shown that endometriosis is progressive in 50% of the cases, early diagnosis is the key [47].

Endometriosis can be classified into three types, each of which affects a different region or tissue of the abdomen. Ovarian endometriosis and peritoneal endometriosis appear as multiple lesions of the endometrial tissue on the ovaries or peritoneum, respectively. Endometriosis of the rectovaginal septum is a more invasive form of endometriosis which involves the infiltration of adenomyotic tissue, making this a more persistent form of the disease [8]. Each of the subtypes has its unique identity due to differences in presentation and underlying pathology. The link between endometriosis and infertility is not completely clear yet. Different reasons have been proposed that could be responsible for infertility such as changes in the peritoneal fluid content and alterations of granulosa cells as well as changes in the follicular fluid including immunological changes.

The high prevalence of ROS in endometriosis patients, due to the oxidant-antioxidant imbalance mentioned previously, results in DNA damage of the sperm that is incubated with peritoneal fluid from endometriosis patients. Although regulated by different factors, the oocyte DNA exhibits similar changes when subjected to incubation with peritoneal fluid from endometriosis patients. Alterations in oocyte DNA and oocyte competence were seen when coincubated with peritoneal fluid from endometriosis patients. Change in endometrial receptivity also plays a role in the resulting infertility in women with endometriosis [67].

The search for molecular markers to identify the symptoms of endometriosis early on in disease development continues, as it will not only aid in preventing progression of the disease but also improve the quality of life, fertility status, and health of women suffering from endometriosis worldwide [68]. Numerous proteins have been identified in different studies (Table 1) and this review will only focus on a few that are either linked to or altered by oxidative stress. According to Fassbender et al., different proteins are expressed at different stages of the menstrual cycle depending on the severity of endometriosis [47].

Seeber et al. were able to identify and validate differentially expressed proteins in the plasma of endometriosis patients. The use of highly accurate methods such as SELDI-TOF-MS may help to potentially avoid laparoscopic procedures, unless absolutely required [48]. In one study, the levels of fibrinogen B peptide were compared in women who underwent laparoscopic procedures with or without pelvic pain. The samples studied were from women in the luteal phase of the menstrual cycle. Fibrinogen B peptide (Table 1) was decreased in endometriosis patients when compared to the control group suggesting its potential use as a biomarker [47]. The conversion of fibrinogen into fibrin is one of the several steps in the blood clotting cascade, after vascular injury. Fibrinogen is also involved in cellular processes, inflammation, and wound healing. It was postulated that the decrease of fibrinogen B chain in the plasma of endometriosis patients indicated that more fibrinogen was converted into fibrin in the peritoneal fluid that led to adhesion and attachment of peritoneal tissue—a hallmark of peritoneal endometriosis [47].

Afamin is a vitamin E-binding protein commonly present in peritoneal fluid. Vitamin E, a nonenzymatic antioxidant, present in nonvascular fluid was found to be decreased in peritoneal fluid of endometriosis patients [74]. Several experiments have shown an increase in levels of Afamin in peritoneal fluid of patients with endometriosis when compared to women who are disease free [8, 74]. Higher level of the binding protein Afamin suggests reduced amounts of available antioxidant to scavenge the free radicals and thereby provides a linkage between endometriosis and oxidative stress.

Iron and its deposits, such as heme and hemosiderin, are typically found in endometriotic lesions [69]. Hemopexin, a heme binding protein, was found to be downregulated in peritoneal fluid of patients proven to have endometriosis [8]. Heme, a breakdown product of erythrocytes, is a source of lipid radicals as it interacts with the lipids present within the plasma membrane, leading to an increase in oxidative stress. Heme is also the source of redox imbalance at the surface of peritoneal epithelial cells [10]. The decrease in hemopexin can therefore lead to oxidative stress as noted in patients of endometriosis. Such radical imbalance also leads to damage in the fallopian tubes and retrograde menstruation, further establishing the role of oxidative stress in the disease [10].

Superoxide dismutase (SOD) is an enzymatic antioxidant produced by the theca interna of ovarian follicles. It catalyzes the breakdown of superoxide radical into hydrogen peroxide and oxygen thereby producing less reactive molecules. Prieto et al. showed that this protein was decreased in the follicular fluid of women with endometriosis (*P* = 0.05), further highlighting the relation between oxidative stress and the disease [31]. SOD is only present in ovaries, particularly in the follicular fluid, providing protection for the theca interna. The follicular fluid is located in a compartment surrounded by highly vascularized cells that act as a blood-follicle barrier [75]. The microvasculature within the theca interna layer is responsible for transferring the SOD from within the cells to the follicular fluid; hence the cells have a protective role.

Thioredoxin (TRX) (Table 1) regulates the levels of oxidative stress in a cell due to its antioxidant activity. It is also involved in multiple cellular processes including proliferation and apoptosis. Thioredoxin binding protein 2 (TBP-2) regulates the activity of thioredoxin and enhances apoptosis in cells with high oxidative stress. Seo et al. noted that there is an increase in TRX/TBP cellular content in endometriosis patients (*P* < 0.001). The study included 66 women (35 with confirmed endometriosis and 31 patients without endometriosis as a control) in which real-time PCR and immunohistochemistry were used for amplification and localization of the proteins in the endometrial tissue. The levels of TBP and TRX in the serum and peritoneal fluid were determined using ELISA. It was postulated that endometriosis related oxidative stress resulted in a decrease in TBP-2 rather than an increase in TRX and hence the change in the TRX/TBP ratio [70]. The decreased negative regulation of TRX was indicative of lower antioxidant activity and increased oxidative stress. The lower levels of TBP-2 in endometrial cells also indicated lower levels of apoptosis and higher levels of cell proliferation. As a result, uterine cells were implanted in different regions leading to the establishment and progression of the endometriosis [70].

Vascular endothelial growth factor (VEGF) is a polypeptide responsible for blood vessel formation by binding to protein kinase receptors. VEGF has an important role in normal angiogenesis as well as in diseased conditions associated with abnormal vascular proliferation [76]. The levels of VEGF increase in the endometrial tissue of women with endometriosis. Furthermore, levels of VEGF differed at different times of the cycle, being higher during the proliferative phase than the secretory phase in patients with endometriosis as compared with women without endometriosis (control). The level of VEGF in the peritoneal fluid was determined using ELISA. VEGF allows the establishment and proliferation of endometriotic tissue as the polypeptide induces neo-vascularization within and around the tissue [72]. Better understanding of this pathway could potentially assist in treatment options as well as lead to the identification of a biomarker of endometriosis. Antiangiogenic factors have been proposed as therapeutic agents for treatment of women suffering from endometriosis. Antiangiogenic drugs function by inhibiting formation, maintenance, and development of blood vessels and therefore act in hindering the progression of endometriosis [77]. Although studies on the effect of oxidative stress on VEGF in the human endometrium have not been done, the relationship has been studied in various other systems. Studies in gastric cancer by Schäfer et al. have demonstrated that an increase in oxidative stress leads to an increase in VEGF gene expression. Moreover, eliminating reactive oxygen species in turn leads to a decrease in VEGF levels proving the specificity of oxidative stress reactions [78]. The effect of oxidative stress on VEGF has also been studied in embryonic development. ROS was noted to cause changes to transcription factors and hence the pathways leading to increased expression of VEGF and angiogenesis for cell survival [79]. Oxidative stress therefore affects VEGF expression but the link to endometriosis is not clear yet.

The ectopic endometrium found in women with endometriosis is an invasive tissue requiring cytoskeletal rearrangement and modification [73]. Beta-actin (Table 1) is one such cytoskeletal protein which is a mediator of cell motility and seems to be increased in the serum of women with endometriosis [73]. Such findings lend credibility to the rationale that endometrial cells change location and shape in order to migrate to their new habitats in the diseased state. Other cytoskeletal proteins that have been identified in the tissues of women with endometriosis include alpha-actinin, ezrin, and talin all of which are decreased in the diseased state. All these proteins are involved in cell adhesion and hence in the binding of cells. The downregulation of these proteins is indicative of changes in cellular morphology and the possible movement of cells away from each other [80].

In addition to cytoskeletal changes within the cell, endometriosis is related to the dynamic changes in the extracellular protein expression. The extracellular matrix (ECM) plays an essential role in some of the cell processes such as migration, adhesion, proliferation, and differentiation as well as acts as the basis for the generation of endometriotic tissue [81]. According to Harrington et al., laminin, fibronectin, collagen IV, and vitronectin, all components of the ECM, were expressed with similar patterns in the endometrial tissue of women with no endometriosis but with a regular menstrual cycle and women exhibiting endometriosis. However, Western blotting techniques were only able to detect a change in tenascin which was upregulated in endometriotic tissue as well as the proliferative phase of the menstrual cycle. Although the exact function of this protein is not known, the change in its levels suggests that the higher expression is a result of the factors present in the peritoneal fluid surrounding endometriotic tissue which may aid in the development of endometriosis [81]. In contrast, increase in vitronectin was seen in the peritoneal fluid of patients with peritoneal and ovarian endometriosis [8]. An increase in fibronectin, collagen IV, and laminin was also seen in women with endometriosis. The release of these molecules into the peritoneal cavity during ovulation may result in increased adhesion of endometrial cells that enter the peritoneal cavity due to retrograde menstruation [82]. Furthermore, fibronectin and collagen type IV of the ECM have been shown to enhance cell proliferation [83, 84]. Increase of vitronectin and tenascin in certain cancers led to the postulation that the progression of endometriosis was similar to tumor formation. Studying ECM proteins and their differential expression was proposed to assist into further understanding the etiology of this enigmatic disease [82].

Cyclins are positive regulators of the cell cycle leading to the proliferation of cells. Their activity is associated with that of cyclin-dependent kinases (CDKs) to keep the cell cycle under control. Cyclin A1 was found to be expressed in the testis, promyelocytic cells, and myeloblastic leukemia [85]. In addition, cyclin A1 was found to be differentially expressed in the serum of patients with endometriosis. The association of such a finding with the pathogenesis of the disease is not well established and requires further studies [73]. It has been postulated that in the presence of pathogenic conditions such as endometriosis, there is an increase in oxidative stress which in turn causes damage to the cell cycle control and leads to excessive proliferation of endometrial stromal cells [86].

Heat shock proteins (HSP) accumulate in cells exposed to stressful conditions. They function as cellular chaperones and play an important role in protein folding/unfolding, transportation and assembly of proteins, and cell cycle control and signaling as well as modulation of apoptosis. HSPs are classified according to their molecular weight [87]. HSP 90 modulates the function of some sex steroid receptor proteins. An increase was reported in the levels of HSP 90 during the proliferative phase of the menstrual cycle detected by staining for the protein in the endometrial glands of a normal endometrium retrieved from women who underwent hysterectomy as treatment for cervical intraepithelial neoplasia. This increase mirrored the change occurring at the hormonal level [88]. There are discrepancies reported in the expression levels of HSP90 in endometriosis. While Matsuzaki et al. reported an increase in HSP90 expression, Fowler et al. noted a decrease in the level of the protein in endometrial tissue [12, 89]. The role of HSPs in correcting the damage occurring in the cell allows these small molecules to counteract the effect of oxidative stress and hence potentiates their use as therapeutic agents for various diseases [90]. This theory further correlates with the effect of oxidative stress, as well as the decrease of HSP90 during the progression of endometriosis.

The above proteins are only a sample of the diverse reactions and changes that occur during endometriosis. Antioxidant proteins are underexpressed while those inducing oxidative stress appear to be overexpressed in multiple studies, corroborating the theory that the imbalance of radicals within the body could potentially cause the endometriosis. While levels of Afamin (an antioxidant binding protein) increased in the peritoneal fluid of patients, other authors have shown a decrease in the expression of some proteins that inhibited oxidative stress formation such as hemopexin [10, 74]. Studies from different environments also revealed differential protein expression such as decrease in SOD levels in follicular fluid and an increase in the TRX/TBP ratio in endometrial cells [31, 70]. Proteins with roles secondary to the function of oxidative stress were also altered, including those that cause neovascularization (VEGF) and cytoskeletal proteins that affect cell division, motility, and differentiation [72, 80]. In addition, proteins of the ECM and cell cycle regulators seem to alter the way cells proliferate and may lead to endometriosis [73]. Heat shock proteins, responsible for the circumventing the effects of oxidative stress, are reported to decrease in endometriosis allowing for more cellular damage [12].

## 6. Role of Proteomics in Polycystic Ovarian Syndrome (PCOS)

PCOS is one of the major causes of infertility and is reported to affect 5–10% of women during their reproductive age [91]. In 1990, National Institute of Health defined polycystic ovarian syndrome (PCOS) as a disease featuring both chronic anovulation and clinical or biochemical signs of hyperandrogenism, that are definitely not due to any other etiology. The presence of polycystic ovaries became one of the required observations for accurate diagnosis of PCOS. It was later noted that PCOS is a symptom of ovarian dysfunction rather than a diagnosis on its own [92]. The Rotterdam diagnostic criterion for PCOS states that two out of the three listed criteria should be present in order to diagnose a woman with this condition:

1. clinical hyperandrogenism (Ferriman-Gallwey score >8) or biochemical hyperandrogenism (elevated total/free testosterone);
2. oligomenorrhea (<6 menstrual cycles per year) or oligo-ovulation;
3. polycystic ovaries on ultrasound (≥12 antral follicles in one ovary or ovarian volume ≥10 cm³) [92].

Hyperandrogenism is commonly seen in PCOS patients due to the elevated LH levels [93]. The condition most commonly manifests itself in the form of hirsutism and acne which may eventually lead to social and psychological distress [91]. 50% of PCOS patients are obese, mostly presenting with an abdominal fat phenotype. This metabolic syndrome eventually leads to the manifestation of different pathologies including diabetes and cardiovascular disease [94]. PCOS patients are commonly found to be insulin-resistant, irrespective of their weight or infertility, and exhibit hyperinsulinemia.

Infertility is an associated problem in patients with PCOS, directing many of the women to seek help through assisted reproductive techniques (ART). According to the World Health Organization (WHO), women with ovulatory disorders, including PCOS, can be successfully treated with ovulation induction along with timed intercourse or any other form or assisted fertilization [4]. Due to the increased risk of ovarian hyperstimulation syndrome and multiple pregnancies, intrauterine insemination (IUI) is the least favored method of ART that is adopted for PCOS patients. Instead, IVF has become the more effective option for women with PCOS to become pregnant [4].

PCOS was thought to be due to genetic predisposition, including evolutionary and environmental modifications. This in turn led to genetic studies of women with PCOS in comparison with disease-free controls but no definitive answers were obtained [95]. The lack of any conclusive information from genetic studies led scientists to focus more on the protein complements instead. The ability to identify certain molecules that are characteristically under-or overexpressed in PCOS patients compared with controls could provide additional insights into the disease. In addition to better understanding of the disease, advanced tools including proteomics will also provide physicians with an excellent tool for diagnosis of a disease that is currently difficult to diagnose, due to multiple manifestations. It would also aid in the identification of therapeutic agents and newer treatment modalities [96]. By examining the proteins, researchers may also have insight into the biological pathways involved with PCOS. This could eventually help in the identification of changes occurring due to oxidative stress or different environmental factors.

Proteomic studies for PCOS have been conducted on different body fluids and tissues including ovarian tissue, serum, visceral adipose tissue, plasma, and ovarian granulose cells. Follicular fluid has been utilized extensively due to its easy accessibility by transvaginal ultrasound-guided oocyte retrieval, following hormonal treatment. The vascularization of the theca interna allows the transduction of different factors from circulation transudating into the follicular fluid, thus providing a representative study medium [38]. Follicular fluid contains metabolic products of granulosa cells, proteins from the follicle and blood, hormones, growth factors, and stem cells and will thereby provide information about the function of each of these molecules in the process of folliculogenesis [14].

Dai and Lu were able to identify 20 differentially expressed proteins when comparing PCOS patients and normal women. The proteins were involved in glucose metabolism, lipoprotein metabolism, cell proliferation, apoptosis, and insulin resistance [14]. While Cortón et al. were able to identify only 15 differentially expressed proteins, Ma et al. identified 69 proteins [6, 13]. Several other studies were also able to identify differentially expressed proteins in different testing media. A list of the more common proteins is presented in Table 2.

Heat shock proteins (HSP) are low molecular weight molecules that can function at temperatures higher than other proteins. They are usually responsible for maintaining cellular homeostasis and act as chaperones of proper protein folding. According to Ma et al., HSP27 (Table 2) is highly expressed in normal ovarian tissue compared to tissue from PCOS patients. In this study, only women in their follicular phase, younger than 30 years with regular menstrual cycles, were selected as controls. Patients with PCOS were only included if they had >12 follicles in one ovary or 2 of the 4 criteria: irregular menstrual cycles, polycystic ovaries, hyperandrogenism, and chronic anovulation [13]. This protein is responsible for maintaining proper protein shape and configuration and acts as an antioxidant in cells. In stressful cellular environments, HSP27 expression was shown to be elevated in order to confer resistance against oxidative stress [97]. HSP27 was also shown to suppress apoptosis through different methods, either by inhibiting cellular caspases, deactivating the ROS in the cell, or blocking Fas-induced apoptosis. The downregulation of this protein indicated lower levels of antioxidants in PCOS ovaries, implying the involvement of oxidative stress underlying PCOS and infertility [13].

Iron is a heavy metal capable of having multiple oxidative levels and therefore acts as a redox molecule. The ability of iron to transfer electrons endows it with the power of forming free radical molecules. Free iron catalyzes the formation of hydroxyl radicals by the Fenton/Haber-Weiss reaction, requiring iron to remain bound within solutions. Transferrin is an iron-binding protein present in different biological fluids [98]. An increase in free iron is thought to cause oxidative stress within cells with concurrent increase in ROS levels. Women with PCOS are thought to exhibit more oxidative stress in follicular fluid [38]. This, in turn, would mean lower transferrin levels within follicular fluid. On the contrary, Dai and Lu found increased levels of transferrin in follicular fluid during controlled ovarian hyperstimulation [14]. It was postulated that this was due to the high levels of transferrin that inhibited FSH from binding to granulosa cells. The diminished levels of FSH, binding to granulosa cells, result in altered follicular growth and maturation as proposed by Kawano et al. [99].

Apolipoprotein A1 (APOA1) is a component of high-density lipoprotein (HDL), proven to maintain the structure and function of the lipoprotein. Apolipoprotein A4 (APOA4) is also a member of the apolipoprotein family with the weakest lipophilic ability. Dai and Lu showed that both APOA1 and APOA4 were significantly increased in the follicular fluid of PCOS patients when compared with patients without PCOS, proving their role in lipid metabolism and abnormal ovulation. These proteins were identified by 2D-GE and MALDI-TOF and confirmed by Western blotting [14]. Lipid metabolism may eventually lead to the formation of lipid peroxides that contribute to the ROS content of a cell. It was shown that lipid peroxides are usually associated with HDL and not LDL, suggesting their association with APOA1 [100, 101]. Such indications led researchers to postulate the role of APOA1 as a biomarker for PCOS due to its presence in plasma and follicular fluid making it an easy target to assess.

Peroxiredoxin (Prx) and glutathione S-transferase M3 (GSTM3) are both antioxidant proteins that were significantly expressed in adipose tissue of PCOS patients. The relation of oxidative stress with PCOS was evident through the differential expression of these proteins between control subjects and PCOS patients in a study done by Cortón et al. The study included 19 morbidly obese women who were undergoing bariatric surgery. 10 of these women were diagnosed with PCOS. 9 women included in the study as controls exhibited no signs of hyperandrogenism and had regular menstrual cycles. The diagnosis of PCOS was based on the presence of oligo-ovulation, hyperandrogenism, and hirsutism, excluding hyperprolactinemia, congenital adrenal hyperplasia, and any androgen secreting tumors [6]. Interestingly, Prx was decreased as GSTM3 increased, although they both function as antioxidants within the cell. As the cells possess lower antioxidant capacity, due to a decrease in Prx, they become more susceptible to oxidative stress. In an attempt to correct for this imbalance, adipose cells produce more GSTM3 which explains the discrepancy between the expression levels of these two proteins. GSTM3 also functions in the breakdown of cytotoxic cellular components which, in turn, leads to an increase in oxidative stress levels in the cell [6]. Therefore, the difference in expression between the two proteins may appear to work in opposite directions when in fact over- and underexpression of each of these proteins lead to the same result: increase in oxidative stress due to decrease in antioxidant activity and the breakdown of cytotoxic cell components.

In the same study by Cortón et al., it was also noted that the expression of annexin V increased in the adipose tissue of women with PCOS [6]. Several functions have been attributed to the annexin family of proteins. They appear to function in regulation of membrane organization and may also be involved with apoptosis [102]. Leon et al. showed that annexin V in particular was involved in a series of cascade reactions that in turn led to the binding of the protein to the interferon-gamma receptor [103]. Interferon-gamma is a cytokine responsible for the production of ROS in the body and in this case explains the increase in annexin V and its significance is demonstrated by increased levels of ROS in PCOS patients.

The extracellular matrix (ECM) and the cellular environment are critical players in PCOS pathology. The ECM is the microenvironment around cells that provides them with physical support. It is also responsible for transmitting signals for the different cellular functions [37]. Matrix metalloproteinases (MMP) are extracellular peptidases that hydrolyze several proteins including those making up the ECM and therefore could contribute to the altered cellular environment and various pathologies [104]. Prolidase is one such MMP involved in collagen remodeling and cell growth and its activity was found to be correlated with oxidative stress levels in the body [105]. Hilali et al. studied the amount of prolidase expressed in PCOS patients in an attempt to assess the association of MMP with the disease. The results showed that prolidase was increased in the serum of PCOS patients in comparison to normal women in addition to the increase in oxidative stress levels [37]. Multiple studies reported increased prolidase activity in patients with cardiovascular disease [106]. The increased degradation of the ECM can also be a causative agent of PCOS, possibly due to the remodeling of ovarian tissue structure and that could subsequently lead to the condition of polycystic ovaries. Different MMP molecules are already being used as biomarkers for the detection of different cardiovascular diseases [106]. Increased activity of prolidase is almost always accompanied with oxidative stress in the pathology of PCOS; they may be used in combination with other clinical factors as biomarkers for early and accurate diagnosis of PCOS in women where diagnosis has been difficult.

## 7. Role of Proteomics in Unexplained Infertility

Unexplained infertility is a diagnosis made by exclusion when no other factors seem to contribute to this pathophysiology [39]. Although no definitive cause of infertility in cases of idiopathic infertility has been established yet, several studies have linked the diagnosis of unexplained infertility with an increase in oxidative stress and the converse where there is an improvement in the fertility status following antioxidant supplementation also holds true [39]. The infertility diagnosis in women is generally established after ruling out male factor, ovulatory dysfunction, hormonal causes, and other known causes of infertility. According to the study performed by Smith et al., 30% of infertile couples are labeled as having unexplained infertility. One of the major contributing factors is usually the age of the female partner [107].

Gleicher and Barad suggested that unexplained infertility is not a diagnosis but is the lack of one and therefore impossible to prove. They argued that the use of the word “unexplained infertility” is inaccurate as it would mean that all of the reproductive workup has been done, when in fact there is no definitive answer as to what represents a complete reproductive workup [108, 109]. They go on to explain the different reasons that could cause infertility that are not usually checked by physicians such as premature aging of ovaries, autoimmune disease, or even misdiagnosed tubal disease [109].

Endometrial receptivity is another cause of infertility that clinicians are currently unable to characterize with definitive markers [44]. The endometrium is receptive for only 4-5 days of the menstrual cycle allowing a narrow window for embryonic implantation. During this time, the cells become secretory and release several molecules that help in implantation and embryo development in what is called the window of implantation [110]. It is important that physicians are able to identify such molecules in the uterine fluid and tissue, within that time frame, in order to provide a noninvasive method to check for the appropriate environment for establishing a pregnancy. Implantation failure is currently the most common limiting factor in achieving pregnancy in women undergoing ART. Therefore, establishing endometrial receptivity is crucial for improved outcomes with ART [111]. Several reports indicate that genes expressed at different stages of the endometrial cycle do not adequately represent the protein expression during that time and hence the need for proteomics for the identification of potential biomarkers of endometrial receptivity [112]. Some of the proteins identified are listed in Table 3.

In the experiment performed by Domínguez, both annexin A2 and S100-A10, components of the annexin heterotetramer (A11t), were upregulated in the endometrial tissue obtained during the prereceptive and receptive phase of the menstrual cycle. These protein measurements were conducted by differential in-gel electrophoresis and MALDI-MS, during the endometrial receptive phase. These proteins were shown to be associated with higher chances of implantation [113]. Annexin A2 was originally thought to function in cell-cell interaction and adhesion. However, different biochemical alterations, such as glycosylation and glutathionylation, may cause functional alterations secondary to changes in the structure. Glutathionylation seems to occur with development of oxidative stress. The changes incurred on a protein due to either glutathionylation or glycosylation may be inhibitory or stimulatory. Glutathionylation of A11t causes a decrease in the protein's biological function. Both annexin A2 and S100-A10 activity were reduced in cases with higher levels of oxidative stress [120]. A decrease in annexin concentration could in turn inhibit cellular reorganization in endometrial cells. The lack of annexin A2 during the receptive phase is one example where oxidative stress may alter protein function and structure leading to detrimental effects at the cellular level. Without the remodeling of endometrial cells and cell-to-cell dialogue, pregnancy outcomes decrease with disruption of implantation.

## 8. Proteomics and ART

The promise of assisted reproductive techniques to achieve pregnancy has led numerous infertile couples to opt for the procedures, with the hope of fulfilling their dream of having their own biological child. It is, however, noted that these techniques are not always associated with higher clinical pregnancy and live birth rates. To achieve a high pregnancy rate it is important to understand the proteins involved in follicular microenvironment [117]. Women have to undergo controlled ovarian stimulation to isolate mature oocytes for the use in IVF or ICSI procedures. In an ART setting, the effects of oxidative stress are amplified due to the lack of internal defense mechanisms that would normally operate* in vivo* as well as increased levels of ROS originating as a result of manipulation of male and female gametes [121]. The high ROS levels may impact the energy pathways in the newly formed embryo and impair embryonic development [121].

Oxidative stress levels were studied by Younis et al. in women undergoing ovarian stimulation prior to ART. In the study by Younis et al., it was noted that the level of antioxidant proteins increased during the peak estrogen levels [9]. The increase in superoxide dismutase (SOD), paraoxonase 1 (PON1), and interleukin 6 (IL6) in plasma correlated positively with pregnancy outcomes and was therefore thought to have potential use as biomarkers for predicting positive pregnancy (Table 3) [9]. Prieto et al. showed that the increase in SOD levels in follicular fluid was predictive of successful pregnancies in IVF patients; hence it could potentially be utilized as a biomarker [31]. Both PON1 and SOD are enzymatic antioxidants present in the blood and their upregulation during ovarian stimulation highlights the role of oxidative stress in reducing pregnancy rates. However, this is in contrast to other studies that showed a higher amount of oxidative stress during ovarian stimulation with a concomitant reduction in antioxidant proteins [100].

Jarkovska et al. conducted a study in which they compared the protein expression within follicular fluid and plasma in women undergoing ovarian stimulation. It was reported that the immunological pathways are one of the major determinants of successful IVF rates. A regulated complement system seems to be essential for proper implantation and embryo maturation during pregnancy with successful IVF procedures. The activity of the complement system decreased VEGF activity leading to less blood vessel formation within the placenta. The inappropriate complement activity is also directly related to a higher rate of abortions. In addition, proteins involved with angiogenesis and blood coagulation seem to be essential in achieving a successful pregnancy following IVF. The list of proteins identified for optimal embryo implantation included antithrombin III precursor, complement precursors, and fibrinogen beta chain precursor (Table 3) [117]. As noted earlier, VEGF levels increase in response to higher levels of oxidative stress further implicating that ROS has a negative effect on ART success rates [72].

The levels of complement C3 and antithrombin were decreased in the follicular fluid in a group of young women who had successful live births in comparison with those who underwent IVF procedures with no success. In addition, higher levels of fibrinogen alpha chain were associated with increased likelihood of live births [122]. Fibrinogen levels in the peritoneal fluid were previously highlighted in this review to serve as potential biomarkers for the presence of endometriosis [47]. Higher levels of fibrinogen may be an indicator of the hypercoagulable state of pregnancy as higher levels of fibrinogen have been reported along with a decrease in fibrinolysis [123]. The altered expression of these various proteins and their successful reproducibility in multiple experiments reinforce their potential role as a diagnostic tool in infertility and ART.

## 9. Protein Profiles of Embryos in ART 

In addition to its implementation in analyzing success rates of IVF, proteomics has been used to study the secretome of embryos in the hope of discovering a biomarker to assess and predict embryo viability. However, the knowledge on preimplantation embryo is currently limited due to technical difficulties. Restrictions such as limited template of known proteins, low quantity, and lack of sensitivity of equipment are all impediments that should be overcome before definitive studies can be accomplished [15]. Embryo selection in ART is currently being done using morphology as the only determinant of embryo viability. Such methods present with several limitations and most importantly, its lack of representation of genomic differences [4]. Several studies have looked at the protein profile of embryos and their secretome in order to determine a suitable marker for implantation success. Some of the proteins identified are listed in Table 3.

Katz-Jaffe et al. identified multiple differentially expressed proteins between blastocysts that matured or did not and blastocysts at different developmental stages as well as embryos that were morphologically similar but did not necessarily possess the same protein profile (listed in Table 3) [118]. Embryos that failed to develop were shown to have higher levels of apoptotic and growth inhibiting proteins including cystatin-like precursor protein which functioned in inhibiting cysteine proteases [118]. Protease activity is crucial in the degradation of the extracellular matrix in the uterine environment and therefore in implantation [124]. The high expression level of cystatin-like precursor protein can function as a potential biomarker for successful embryo implantation and help select the embryos with the highest chance of implantation. Although not proven in embryos, studies on rat CNS neurons have shown that an increase in oxidative stress eventually leads to an increase in cystatin C activity. This may therefore explain why an increase in oxidative stress* in utero* is linked to lower fertility status [125].

Katz-Jaffe et al. found that the proteins secreted from the embryo over a period of 24 hours were highly reflective of what was going on inside the cells, as proteins are the major determinants of cell function [119]. Ubiquitin, a polypeptide that functions as part of the protein degrading complex, was found to be differentially expressed in different cells. Significantly higher levels of the protein were observed in developing blastocyst secretome but seemed to be dminished in the secretome of degenerating embryos [119].

The ubiquitin-proteosome pathway (UPP) was shown to be involved in uterine remodeling and placental development which allows successful implantation. Wang et al. were able to prove that blocking this pathway inhibited pregnancy in different species including humans [126]. The change of ubiquitin expression in the uterine tissue also altered the activity of the matrix metalloproteinases which are important during the early pregnancy [126, 127]. Ubiquitin levels measured in the secretome of the embryo may therefore be used as a biomarker for noninvasive and easy prediction of successful pregnancy.

Proteomic studies on the embryo are still limited due to experimental difficulties especially in human embryos. Finding a greater number of differentially expressed proteins will increase our knowledge on the activity of embryos and what is essential for successful implantation and pregnancy. These proteins may also be used as biomarkers to select the embryos before transferring them during ART. This can help avoid unsuccessful transfers as well as the transfer of karyotypically abnormal embryos.

## 10. Conclusion 

In this review, we have highlighted studies that have identified some of the proteins in female infertility-related diseases and their association with underlying oxidative stress. Endometriosis, PCOS, and unexplained infertility are currently the three most common causes of infertility among women worldwide. Understanding of these diseases will provide clinicians with a better insight into their early diagnosis and management. Identification of proteins in different diseases and at different stages of the progression will help in the discovery of potential biomarkers for noninvasive diagnosis of these diseases. The various proteins listed in the review have been separated, identified, and confirmed using 2D-GE, mass spectrometry, and Western blotting, respectively. 2D- PAGE is beneficial in its ability to detect proteins with wide range of masses and identify several proteins at any one time. However, it is a time-consuming process and requires extensive laboratory skills. Mass spectrometry has the advantage of being rapid and can utilize even a small sample size but is limited by the availability of test samples that can be used. Western blotting is characterized by its high sensitivity and specificity; however, some drawbacks in the technique include its high technical demand and being prone to false results. In addition, we looked at proteins identified in the embryonic secretome. Definitive knowledge of the embryonic secretome can assist in the selection of the best embryo and help clinics to move towards single embryo transfers without any impact on the success rates.

Future research should be directed to identify specific proteins in the abovementioned diseases as well as proteins related to identification of best embryos. Researchers should also aim to achieve reproducible results and validate these proteins as this is a main requirement in biomarker credibility. The ability to identify a protein, common between women of different races, ages, and in different regions of the world, will hopefully contribute to improving women's health worldwide.

## Figures and Tables

**Table 1:** Proteins identified in relation to endometriosis.

Protein assessed/technique of assessment	Function and site of expression	Source	Association with endometriosis	Population size	Reference
Fibrinogen B peptide/SELDI-TOF-MS	Helps in blood coagulation, regulation of cell adhesion, and spreading.	Plasma	Decreased in endometriosis due to overconsumption and the consequence is adhesion of endometrial fragments.	254 samples from women who underwent laparoscopy with or without pelvic pain. 89 women without endometriosis and 165 with endometriosis	[47]

Afamin/ELISA or 2D-gel electrophoresis	Vitamin E binding protein.	Peritoneal fluid	Increased causing a decrease in antioxidant activity	242 reproductive age women. [65] 24 samples of peritoneal fluid from symptomatic women [8]	[8, 69]

Hemopexin/2Dgel electrophoresis	Heme binding protein	Peritoneal fluid	Decreased leading to oxidative stress	24 samples of peritoneal fluid from symptomatic women	[8]

Inter-alpha inhibitor H4 (ITIH4)/2Dgel electrophoresis	Not clear but involved in acute phase inflammation	Peritoneal fluid	Increased in peritoneal endometriosis.	24 samples of peritoneal fluid from symptomatic women	[8]

Apolipoprotein A4/2Dgel electrophoresis	Bind lipids to form lipoproteins.	Peritoneal fluid	Decreased in ovarian endometriosis	24 samples of peritoneal fluid from symptomatic women	[8]

Haptoglobin/2Dgel electrophoresis	Hemoglobin binding protein	Peritoneal fluid	Increased. Removes iron and prevents oxidative stress	24 samples of peritoneal fluid from symptomatic women	[8]

Vitronectin/2Dgel electrophoresis	Involved in cell migration, adhesion, and invasion.	Peritoneal fluid	Increased	24 samples of peritoneal fluid from symptomatic women	[8]

SERPINA/2Dgel electrophoresis	Associated with inflammatory outcomes	Peritoneal fluid	Increased and might be a good biomarker	24 samples of peritoneal fluid from symptomatic women	[8]

Superoxide dismutase (SOD)/mass spectrometry, high performance liquid chromatography, and ELISA	Breakdown of superoxide radical into hydrogen peroxide and oxygen	Follicular fluid	Decreased leading to oxidative stress	91 infertile women who were going to undergo IVF, 23 with endometriosis identified by laparoscopy or ultrasound and 68 infertile due to different reasons (not endometriosis)	[31]

Thioredoxin binding protein 2 (TBP)/ELISA	Binds thioredoxin (TRX) which is an antioxidant	Cells from endometriosis tissue	It was noted that there is an increase in the TRX/TBP in endometriosis patients	66 women 35 with confirmed endometriosis and 31 patients without endometriosis as a control	[70]

Interleukins (IL-6, IL-10, and IL-1B)	Function in the immune system of different cells.	Serum and follicular fluid	Increase in follicular fluid showing immune involvement with the disease	38 patients, 20 with endometriosis, and 18 controls	[71]

Vascular endothelial growth factor/ELISA	Increase in vascular permeability	Peritoneal fluid	Increases in patients with endometriosis	43 patients in reproductive age who were undergoing diagnostic laparoscopy for dysmenorrhea or elective laparoscopy for infertility, 19 controls without endometriosis, and 24 patients with endometriosis	[72]

G antigen family B/Mass spectrometry and 2Dgel electrophoresis	Responsible for the androgen-insensitive phenotype	Serum	May effect the level of estrogen and therefore the occurrence of endometriosis	12 women of reproductive age, 6 with laparoscopically proven endometriosis, and 6 without endometriosis	[73]

Carbonic anhydrase 1/mass spectrometry and 2Dgel electrophoresis	Involved with the process of bio-respiration	Serum	Decreased in patients with endometriosis	12 women of reproductive age, 6 with laparoscopically proven endometriosis, and 6 without endometriosis	[73]

Actin family proteins (actin related protein 6 and actin-like-7-alpha)/mass spectrometry and 2D-gel electrophoresis	Actin is in the cellular cytoskeleton and involved in mitosis, signal transduction, and motility.	Serum	Differentially expressed in endometriosis	12 women of reproductive age, 6 with laparoscopically proven endometriosis, and 6 without endometriosis	[73]

CD166 antigen/mass spectrometry and 2D-gel electrophoresis	Cell adhesion molecules that bind to CD6 and might play a role in T and B cell adhesion to leukocytes	Serum	Downregulated causing change in immune function of endometriosis patients	12 women of reproductive age, 6 with laparoscopically proven endometriosis, and 6 without endometriosis	[73]

CyclinA1/mass spectrometry and 2D-gel electrophoresis	Controls the cell cycle at G1/S and the G2/M stops	Serum	Differentially expressed	12 women of reproductive age, 6 with laparoscopically proven endometriosis and 6 without endometriosis	[73]

Vimentin/mass spectrometry and 2D-gel electrophoresis	A component of the cytoskeleton especially in mesenchymal cells	Serum	Increased in endometriosis tissue as cytoskeletal changes are required	12 women of reproductive age, 6 with laparoscopically proven endometriosis, and 6 without endometriosis	[73]

B-actin/mass spectrometry and 2D-gel electrophoresis	Component of the cytoskeleton and mediates cell motility and may affect expression of some endometriosis related genes	Serum	Increased as cellular changes occur	12 women of reproductive age, 6 with laparoscopically proven endometriosis, and 6 without endometriosis	[73]

ATP synthase/mass spectrometry and 2D gel electrophoresis	The production of ATP from ADP	Serum	Increased as retrograde menstruation requires ATP	12 women of reproductive age, 6 with laparoscopically proven endometriosis and 6 without endometriosis	[73]

Glycodelin/2D-gel electrophoresis	Involved in regulating uterine environment for pregnancy and timing the occurrence of fertilization events	Endometrial tissue	Increased in the eutopic endometrium of patients	24 women undergoing laparoscopy either for endometriosis, elective tubal sterilization, or diagnostic purposes.	[12]

HSP90/2D-gel electrophoresis	Chaperone molecule for the correct folding of proteins	Endometrial tissue	Decreased expression in eutopic endometrium	24 women undergoing laparoscopy either for endometriosis, elective tubal sterilization, or diagnostic purposes	[12]

Annexin A2/2D-gel electrophoresis	Involved in the cytoskeleton and exocytosis	Endometrial tissue	Decreased in patients showing less secretory activity	24 women undergoing laparoscopy either for endometriosis, elective tubal sterilization, or diagnostic purposes.	[12]

Peroxiredoxin/2D-gel electrophoresis	Antioxidant molecules	Endometrial tissue	Altered expression in endometrial tissue	24 women undergoing laparoscopy either for endometriosis, elective tubal sterilization, or diagnostic purposes.	[12]

**Table 2:** Proteins differentially expressed in PCOS patients.

Protein and method	Function	Site of expression	Relation to PCOS	Population size	Reference
HSP27/2D gel electrophoresis and mass spectrometry	Steroidogenesis, chaperoning and protection against apoptosis	Ovarian tissue	Decreased	3 normal women and 3 with PCOS all younger than 30 years old	[13]

HSP10/2D gel electrophoresis and mass spectrometry	Protect against apoptosis and play a role in follicular atresia	Ovarian tissue	Decreased	3 normal women and 3 with PCOS all younger than 30 years old	[13]

HSP47/2D gel electrophoresis and mass spectrometry	Collagen specific molecular chaperone involved in fibrolytic disease	Ovarian tissue	Increased	3 normal women and 3 with PCOS all younger than 30 years old	[13]

FUSE binding protein 1/2D gel electrophoresis and mass spectrometry	Regulation of insulin function	Ovarian tissue	Increased	3 normal women and 3 with PCOS all younger than 30 years old	[13]

Glyoxylate reductase/ Hydroxypyruvate reductase/2D gel electrophoresis and mass spectrometry	Regulation of insulin function	Ovarian tissue	Increased	3 normal women and 3 with PCOS all younger than 30 years old	[13]

Antithrombin III/2D gel electrophoresis and mass spectrometry	Regulation of fibrinolysis and thrombosis	Ovarian tissue	Decreased	3 normal women and 3 with PCOS all younger than 30 years old	[13]

Annexin A2/2D gel electrophoresis and mass spectrometry	Regulation of fibrinolysis and thrombosis	Ovarian tissue	Decreased	3 normal women and 3 with PCOS	[13]

Fibrinogen alpha and gamma chain/2D gel electrophoresis and mass spectrometry	Fibrinolysis and blood clotting	Ovarian tissue	Increased	3 normal women and 3 with PCOS all younger than 30 years old with PCOS	[13]

Laminin A/C/2D gel electrophoresis and mass spectrometry	Regulators of the ovary	Ovarian tissue	Increased	3 normal women and 3 with PCOS all younger than 30 years old	[13]

Vimentin/2D gel electrophoresis and mass spectrometry	Regulator of the ovary	Ovarian tissue	Increased	3 normal women and 3 with PCOS all younger than 30 years old	[13]

Vitamin D binding protein (DBP)/2D gel electrophoresis and MALDI-TOF	Binding vitamin D	Follicular fluid	Decreased	30 normal women undergoing IVF and 30 with PCOS	[14]

Plasma retinol binding protein (RBP4)/2D gel electrophoresis and MALDI-TOF	Binding of retinol	Follicular fluid	Increased	30 normal women undergoing IVF and 30 with PCOS	[14]

Transthyretin (TTR)/2D gel electrophoresis and MALDI-TOF	Insulin resistance and obesity	Follicular fluid	Increased	30 normal women undergoing IVF and 30 with PCOS	[14]

Apolipoprotein A1/2D gel electrophoresis and MALDI-TOF	Regulate lipid metabolism and has a function in anti-inflammatory pathways	Follicular fluid	Increased	30 normal women undergoing IVF and 30 with PCOS	[14]

Apolipoprotein A4/2D gel electrophoresis and MALDI-TOF	Weakest lipophilic capacity and associated with lipid metabolism	Follicular fluid	Increased	30 normal women undergoing IVF and 30 with PCOS	[14]

Transferrin/2D gel electrophoresis and MALDI-TOF	Binds iron and transports it. Promote cell growth and development	Follicular fluid	Increased	30 normal women undergoing IVF and 30 with PCOS	[14]

DRAM2/2D gel electrophoresis and MALDI-TOF	Apoptosis	Follicular fluid	Decreased	30 normal women undergoing IVF and 30 with PCOS	[14]

MAK (Male germ-associated kinase)/2D gel electrophoresis and MALDI-TOF	Plays a role in meiosis of cells	Follicular fluid	Decreased	30 normal women undergoing IVF and 30 with PCOS	[14]

Glutathione S-transferase M3 (GSTM3)/2D gel electrophoresis and MALDI-MS	Antioxidant involved in breakdown of cytotoxic material	Omental adipose tissue	Increased	19 obese women undergoing bariatric surgery; 10 with PCOS and 9 nonsymptomatic used as control	[6]

Annexin V/2D gel electrophoresis and MALDI-MS	Phospholipid binding	Omental adipose tissue	Increased	19 obese women undergoing bariatric surgery; 10 with PCOS and 9 nonsymptomatic used as control	[6]

Peroxiredoxin 2/2D gel electrophoresis and MALDI-MS	Antioxidant	Omental adipose tissue	Decreased	19 obese women undergoing bariatric surgery; 10 with PCOS and 9 nonsymptomatic used as control	[6]

Actin/2D gel electrophoresis and MALDI-MS	Cytoskeletal protein and gene expression regulation	Omental adipose tissue	Decreased	19 obese women undergoing bariatric surgery; 10 with PCOS and 9 nonsymptomatic used as control	[6]

TPI1/2D gel electrophoresis and MALDI-MS	interconversion of dihydroxyacetone phosphate and glyceraldehydes-3-phosphate in preparation for glycolysis	Omental adipose tissue	Decreased	19 obese women undergoing bariatric surgery; 10 with PCOS and 9 nonsymptomatic used as control	[6]

Prolidase	Matrix metalloproteinase	Serum	Increased	30 PCOS patients and 28 normal women	[37]

**Table 3:** Proteins identified at different endometrial stages, in IVF and in the embryo.

Protein estimated/proteomic technique	Function	Source	Relation to endometrial receptivity or embryo readiness	Population size	Reference
L-Glutamate NMCA receptor zeta subunit 1/LC-MS/MS	Subunit of a ligand gated channel that is involved in neuron plasticity. Other roles include glutamate mediated toxicity in mitochondria leading to apoptosis	Endometrial tissue	Only expressed in the secretory endometrium	6 samples of endometrial tissue from a tissue bank; 3 from proliferative phase and 3 from secretory phase (as classified by a pathologist)	[46]

FRAT1/LC-MS/MS	Proto-oncogene that activates the WNT pathway. Inhibits apoptosis	Endometrial tissue	Only expressed in the secretory endometrium	6 samples of endometrial tissue from a tissue bank; 3 from proliferative phase and 3 from secretory phase (as classified by a pathologist)	[46]

Myosin light chain kinase 2/LC-MS/MS	Muscle contraction	Endometrial tissue	Only expressed in the secretory endometrium	6 samples of endometrial tissue from a tissue bank; 3 from proliferative phase and 3 from secretory phase (as classified by a pathologist)	[46]

Isopentenyl diphosphate delta-isomerase/LC-MS/MS	Catalyzes a step in the formation of isoprenoids.	Endometrial tissue	Only expressed in the secretory endometrium	6 samples of endometrial tissue from a tissue bank; 3 from proliferative phase and 3 from secretory phase (as classified by a pathologist)	[46]

Macrophage migration inhibitor factor (MIF)/LC-MS/MS	Prevent macrophages from performing their job in the endometrium	Endometrial tissue	Found in all phases of the endometrium	6 samples of endometrial tissue from a tissue bank; 3 from proliferative phase and 3 from secretory phase (as classified by a pathologist)	[46]

Glycodelin/LC-MS/MS	Regulating the uterine cavity for pregnancy	Endometrial tissue	Up-regulated during the secretory phase	6 samples of endometrial tissue from a tissue bank; 3 from proliferative phase and 3 from secretory phase (as classified by a pathologist)	[46]

Stathmin 1/gel electrophoresis and MALDI-MS	Cytoskeleton, intracellular signaling cascade	Endometrial tissue	Decreased during receptive phase	8 fertile women in their reproductive age, during prereceptive and receptive times of the menstrual cycle	[113]

Annexin A2/gel electrophoresis and MALDI-MS	Skeletal development		Increased during receptive phase	8 fertile women, in their reproductive age, during prereceptive and receptive times of the menstrual cycle	[113]

Monoamine oxidase A/gel electrophoresis and MALDI-MS	Electron transport	Endometrial tissue	Increased during receptive phase	8 fertile women in their reproductive age, during prereceptive and receptive times of the menstrual cycle	[113]

Membrane-associated progesterone receptor component 1/gel electrophoresis and MALDI-MS	Signaling	Endometrial tissue	Decreased during receptive phase	8 fertile women in their reproductive age, during prereceptive and receptive times of the menstrual cycle	[113]

Collagen alpha 1/gel electrophoresis and MALDI-MS	Skeletal development	Endometrial tissue	Increased during receptive phase	8 fertile women, in their reproductive age, during prereceptive and receptive times of the menstrual cycle	[113]

Annexin A4/gel electrophoresis and MALDI-MS	Signal transduction	Endometrial tissue	Increased during receptive phase	8 fertile women in their reproductive age, during prereceptive and receptive times of the menstrual cycle	[113]

Vimentin/gel electrophoresis and MALDI-MS	Cytoskeleton	Endometrial tissue	Decreased during receptive phase	8 fertile women, in their reproductive age, during prereceptive and receptive times of the menstrual cycle	[113]

S100-A10/gel electrophoresis and MALDI-MS	Signal transduction	Endometrial tissue	Increased during receptive phase	8 fertile women in their reproductive age, during prereceptive and receptive times of the menstrual cycle	[113]

Sulfur oxide dismutase	Enzymatic antioxidant	Serum	Increased during ovarian stimulation	15 patients undergoing ovarian stimulation who had PCOS, endometriosis, or unexplained infertility and all went controlled ovarian stimulation	[9]

Paraoxonase 1 (PON1)	Enzymatic antioxidant	Serum	Increased during ovarian stimulation	15 patients undergoing ovarian stimulation who had PCOS, endometriosis, or unexplained infertility and all went controlled ovarian stimulation	[9]

Glutathione peroxidase (GPx)	Enzymatic antioxidant	Serum	Increased during ovarian stimulation	15 patients undergoing ovarian stimulation who had PCOS, endometriosis, or unexplained infertility and all went controlled ovarian stimulation	[9]

Interleukin 6	Acts as both an anti-inflammatory and proinflammatory cytokine	Serum	Increased during ovarian stimulation	15 patients undergoing ovarian stimulation who had PCOS, endometriosis, or unexplained infertility and all went controlled ovarian stimulation	[9]

Apolipoprotein A1/Tandem MS	Component of high density lipoproteins and acceptor of extra-hepatic cholesterol	Embryo culture media	Decreased in secretome of embryos	702 samples analyzed from women undergoing IVF or from egg donor recipients irrespective of age (26–49 years) or reason for infertility	[11]

Leptin/RT-PCR	Regulates energy uptake and expenditure	Endometrial culture	Increased in competent embryos	Embryos and granulose cells from 9 patients after ovarian hyperstimulation and artificial insemination. Human adipose tissue was used as control for leptin levels. Human endometrial and placental tissue was also studied	[114]

HOXA10/RT-PCR	Functions in embryo viability	Ishikawa cells which are well-differentiated endometrial adenocarcinoma cell line	Increased in cells exposed to blastocyst media	Ishikawa cells grown with blastocyst cells and ones that were placed with an embryo not yet in the blastocyst stage	[115]

Lipocalin-1/mass spectrometry	Transport proteins	Blastocyst secretions	Used for non-invasive aneuploidy testing	65 couples undergoing infertility treatment. Samples were pooled for MS into a group that had 100% implantation from euploid blastocysts or media of aneuploid blastocysts. IVF media without an embryo were used as control	[116]

Complement precursor proteins/2D gel electrophoresis, 2D HPLC and MALDI-MS	Part of the innate immune system	Human follicular fluid and plasma	Altered expression during IVF procedures	38 women (24–38 years) undergoing stimulation prior to IVF. All the samples were used from women with 100% success of IVF	[117]

Fibrinogen Beta precursor/2D gel electrophoresis, 2D HPLC and MALDI-MS	Involved in blood clotting cascade	Human follicular fluid and plasma	Altered expression during IVF procedures	38 women (24–38 years) undergoing stimulation prior to IVF. All the samples were used from women with 100% success of IVF	[117]

Antithrombin III precursor/2D gel electrophoresis, 2D HPLC and MALDI-MS	Inactivates the coagulation system	Human follicular fluid and plasma	Altered expression during IVF procedures	38 women (24–38 years) undergoing stimulation prior to IVF. All the samples were used from women with 100% success of IVF	[117]

Heparin-binding epidermal growth factor- (EGF-) like growth factor precursor/SELDI-TOF-MS	Cell-to-cell juxtacrine signaling inhibiting growth activity	Human embryonic cells	Increased in embryos that failed to develop	21 blastocysts at different developmental stages that had been cryopreserved. They were graded as early, expanded, or degenerated embryos	[118]

Cystatin like precursor/SELDI-TOF-MS	Inhibits cysteine proteases involved in implantation	Human embryonic cells	Increased in embryos that failed to develop	21 blastocysts at different developmental stages that had been cryopreserved. They were graded as early, expanded, or degenerated embryos	[118]

Caspase 1 precursor/SELDI-TOF-MS	Involved in cell death	Human embryonic cells	Increased in embryos that failed to develop	21 blastocysts at different developmental stages that had been cryopreserved. They were graded as early, expanded, or degenerated embryos	[118]

Cytochrome c oxidase subunit VIIIa 3/SELDI-TOF-MS	Part of the electron transport chain in mitochondria	Human embryonic cells	Increased in embryos that failed to develop	21 blastocysts at different developmental stages that had been cryopreserved. They were graded as early, expanded, or degenerated embryos	[118]

Ubiquitin/SELDI-TOF-MS	Part of the ubiquitin-proteosome pathway involved in degradation of proteins	Mice and human embryo secretome	Increased in embryonic secretome associated with successful pregnancies	2-cell human embryos and mouse embryos. Mouse embryos were collected after hyperstimulation of mouse and fertilization. Human embryos were obtained from cryopreserved embryos	[119]

## References

[1] Bhattacharya S, Johnson N, Tijani HA, Hart R, Pandey S, Gibreel AF (2010). Female infertility. *BMJ Clinical Evidence*.

[2] Zegers-Hochschild F, Nygren K-G, Adamson GD (2006). The ICMART glossary on ART terminology. *Human Reproduction*.

[3] Chachamovich JR, Chachamovich E, Ezer H, Fleck MP, Knauth D, Passos EP (2010). Investigating quality of life and health-related quality of life in infertility: a systematic review. *Journal of Psychosomatic Obstetrics & Gynecology*.

[4] Huang JY, Rosenwaks Z (2012). In vitro fertilisation treatment and factors affecting success. *Best Practice & Research Clinical Obstetrics & Gynaecology*.

[5] Ruder EH, Hartman TJ, Goldman MB (2009). Impact of oxidative stress on female fertility. *Current Opinion in Obstetrics & Gynecology*.

[6] Cortón M, Botella-Carretero JI, López JA (2008). Proteomic analysis of human omental adipose tissue in the polycystic ovary syndrome using two-dimensional difference gel electrophoresis and mass spectrometry. *Human Reproduction*.

[7] Agarwal A, Gupta S, Sekhon L, Shah R (2008). Redox considerations in female reproductive function and assisted reproduction: from molecular mechanisms to health implications. *Antioxidants and Redox Signaling*.

[8] Wölfler MM, Meinhold-Heerlein IM, Söhngen L (2011). Two-dimensional gel electrophoresis in peritoneal fluid samples identifies differential protein regulation in patients suffering from peritoneal or ovarian endometriosis. *Fertility and Sterility*.

[9] Younis A, Clower C, Nelsen D (2012). The relationship between pregnancy and oxidative stress markers on patients undergoing ovarian stimulations. *Journal of Assisted Reproduction and Genetics*.

[10] Wölfler MM, Meinhold-Heerlein IM, Henkel C (2013). Reduced hemopexin levels in peritoneal fluid of patients with endometriosis. *Fertility and Sterility*.

[11] Nyalwidhe J, Burch T, Bocca S (2013). The search for biomarkers of human embryo developmental potential in IVF: a comprehensive proteomic approach. *Molecular Human Reproduction*.

[12] Fowler PA, Tattum J, Bhattacharya S (2007). An investigation of the effects of endometriosis on the proteome of human eutopic endometrium: a heterogeneous tissue with a complex disease. *Proteomics*.

[13] Ma X, Fan L, Meng Y (2007). Proteomic analysis of human ovaries from normal and polycystic ovarian syndrome. *Molecular Human Reproduction*.

[14] Dai G, Lu G (2012). Different protein expression patterns associated with polycystic ovary syndrome in human follicular fluid during controlled ovarian hyperstimulation. *Reproduction, Fertility and Development*.

[15] Katz-Jaffe MG, McReynolds S, Gardner DK, Schoolcraft WB (2009). The role of proteomics in defining the human embryonic secretome. *Molecular Human Reproduction*.

[16] Agarwal A, Gupta S, Sharma RK (2005). Role of oxidative stress in female reproduction. *Reproductive, Biology and Endocrinology*.

[17] Borowiecka M, Wojsiat J, Polac I, Radwan M, Radwan P, Zbikowska HM (2012). Oxidative stress markers in follicular fluid of women undergoing in vitro fertilization and embryo transfer. *Systems Biology in Reproductive Medicine*.

[18] Riley JCM, Behrman HR (1991). Oxygen radicals and reactive oxygen species in reproduction. *Proceedings of the Society for Experimental Biology and Medicine*.

[19] Behrman HR, Kodaman PH, Preston SL, Gao S (2001). Oxidative stress and the ovary. *Journal of the Society for Gynecologic Investigation*.

[20] Robertson G, Leclercq I, Farrell GC (2001). Nonalcoholic steatosis and steatohepatitis. II. Cytochrome P-450 enzymes and oxidative stress. *American Journal of Physiology—Gastrointestinal and Liver Physiology*.

[21] Sugino N, Karube-Harada A, Taketani T, Sakata A, Nakamura Y (2004). Withdrawal of ovarian steroids stimulates prostaglandin F2*α* production through nuclear factor-*κ*B activation via oxygen radicals in human endometrial stromal cells: potential relevance to menstruation. *Journal of Reproduction and Development*.

[22] Chandra NSA, Kesavan S, Agarwal A Significance of oxidative stress on human reproduction. http://www.clevelandclinic.org/reproductiveresearchcenter/docs/agradoc316.pdf.

[23] Fujii J, Iuchi Y, Okada F (2005). Fundamental roles of reactive oxygen species and protective mechanisms in the female reproductive system. *Reproductive Biology and Endocrinology*.

[24] Tamura H, Takasaki A, Miwa I (2008). Oxidative stress impairs oocyte quality and melatonin protects oocytes from free radical damage and improves fertilization rate. *Journal of Pineal Research*.

[25] Revelli A, Piane LD, Casano S, Molinari E, Massobrio M, Rinaudo P (2009). Follicular fluid content and oocyte quality: from single biochemical markers to metabolomics. *Reproductive Biology and Endocrinology*.

[26] Pasqualotto EB, Agarwal A, Sharma RK (2004). Effect of oxidative stress in follicular fluid on the outcome of assisted reproductive procedures. *Fertility and Sterility*.

[27] Wiener-Megnazi Z, Vardi L, Lissak A (2004). Oxidative stress indices in follicular fluid as measured by the thermochemiluminescence assay correlate with outcome parameters in in vitro fertilization. *Fertility and Sterility*.

[28] Jana SK, K NB, Chattopadhyay R, Chakravarty B, Chaudhury K (2010). Upper control limit of reactive oxygen species in follicular fluid beyond which viable embryo formation is not favorable. *Reproductive Toxicology*.

[29] Szczepańska M, Koźlik J, Skrzypczak J, Mikołajczyk M (2003). Oxidative stress may be a piece in the endometriosis puzzle. *Fertility and Sterility*.

[30] Liu Y, Luo L, Zhao H (2001). Levels of lipid perioxides and superoxide dismutase in peritoneal fluid of patients with endometriosis. *Journal of Tongji Medical University*.

[31] Prieto L, Quesada JF, Cambero O (2012). Analysis of follicular fluid and serum markers of oxidative stress in women with infertility related to endometriosis. *Fertility and Sterility*.

[32] Gupta NSS, Metterle L, Chandra A, Agarwal A Antioxidants and female reproductive pathologies. http://ccf.org/reproductiveresearchcenter/docs/agradoc322.pdf.

[33] Jackson LW, Schisterman EF, Dey-Rao R, Browne R, Armstrong D (2005). Oxidative stress and endometriosis. *Human Reproduction*.

[34] Murphy AA, Santanam N, Parthasarathy S (1998). Endometriosis: a disease of oxidative stress?. *Seminars in Reproductive Endocrinology*.

[35] Agarwal A, Gupta S, Sikka S (2006). The role of free radicals and antioxidants in reproduction. *Current Opinion in Obstetrics and Gynecology*.

[36] Seino T, Saito H, Kaneko T, Takahashi T, Kawachiya S, Kurachi H (2002). Eight-hydroxy-2′-deoxyguanosine in granulosa cells is correlated with the quality of oocytes and embryos in an in vitro fertilization-embryo transfer program. *Fertility and Sterility*.

[37] Hilali N, Vural M, Camuzcuoglu H, Camuzcuoglu A, Aksoy N (2013). Increased prolidase activity and oxidative stress in PCOS. *Clinical Endocrinology*.

[38] Chattopadhayay R, Ganesh A, Samanta J, Jana SK, Chakravarty BN, Chaudhury K (2010). Effect of follicular fluid oxidative stress on meiotic spindle formation in infertile women with polycystic ovarian syndrome. *Gynecologic and Obstetric Investigation*.

[39] Agarwal A, Aponte-Mellado A, Premkumar BJ, Shaman A, Gupta S (2012). The effects of oxidative stress on female reproduction: a review. *Reproductive Biology and Endocrinology*.

[40] Polak G, Kozioł-Montewka M, Tarkowski R, Kotarski J (2001). Peritoneal fluid and plasma 4-hydroxynonenal and malonyldialdehyde concentrations in infertile women. *Ginekologia Polska*.

[41] Wang Y, Sharma RK, Falcone T, Goldberg J, Agarwal A (1997). Importance of reactive oxygen species in the peritoneal fluid of women with endometriosis or idiopathic infertility. *Fertility and Sterility*.

[42] Paszkowski T, Traub AI, Robinson SY, McMaster D (1995). Selenium dependent glutathione peroxidase activity in human follicular fluid. *Clinica Chimica Acta*.

[43] Jensen ON (2004). Modification-specific proteomics: characterization of post-translational modifications by mass spectrometry. *Current Opinion in Chemical Biology*.

[44] Meehan KL, Rainczuk A, Salamonsen LA, Stephens AN (2010). Proteomics and the search for biomarkers of female reproductive diseases. *Reproduction*.

[45] Upadhyay RD, Balasinor NH, Kumar AV, Sachdeva G, Parte P, Dumasia K (2013). Proteomics in reproductive biology: beacon for unraveling the molecular complexities. *Biochimica et Biophysica Acta*.

[46] DeSouza L, Diehl G, Yang ECC (2005). Proteomic analysis of the proliferative and secretory phases of the human endometrium: protein identification and differential protein expression. *Proteomics*.

[47] Fassbender A, Waelkens E, Verbeeck N (2012). Proteomics analysis of plasma for early diagnosis of endometriosis. *Obstetrics and Gynecology*.

[48] Seeber B, Sammel MD, Fan X (2010). Proteomic analysis of serum yields six candidate proteins that are differentially regulated in a subset of women with endometriosis. *Fertility and Sterility*.

[49] Görg A, Weiss W, Dunn MJ (2004). Current two-dimensional electrophoresis technology for proteomics. *Proteomics*.

[50] May C, Brosseron F, Chartowski P, Schumbrutzki C, Schoenebeck B, Marcus K (2011). Instruments and methods in proteomics. *Data Mining in Proteomics*.

[51] Swank RT, Munkres KD (1971). Molecular weight analysis of oligopeptides by electrophoresis in polyacrylamide gel with sodium dodecyl sulfate. *Analytical Biochemistry*.

[52] Wilkins MR, Pasquali C, Appel RD (1996). From proteins to proteomes: large scale protein identification by two-dimensional electrophoresis and amino acid analysis. *Biotechnology*.

[53] Bunai K, Yamane K (2005). Effectiveness and limitation of two-dimensional gel electrophoresis in bacterial membrane protein proteomics and perspectives. *Journal of Chromatography B: Analytical Technologies in the Biomedical and Life Sciences*.

[54] Glish GL, Vachet RW (2003). The basics of mass spectrometry in the twenty-first century. *Nature Reviews Drug Discovery*.

[55] Ducret A, van Oostveen I, Eng JK, Yates JR, Aebersold R (1998). High throughput protein characterization by automated reverse-phase chromatography/electrospray tandem mass spectrometry. *Protein Science*.

[56] Blackburn K, Mbeunkui F, Mitra SK, Mentzel T, Goshe MB (2010). Improving protein and proteome coverage through data-independent multiplexed peptide fragmentation. *Journal of Proteome Research*.

[57] Michalski A, Damoc E, Hauschild J-P (2011). Mass spectrometry-based proteomics using Q Exactive, a high-performance benchtop quadrupole orbitrap mass spectrometer. *Molecular & Cellular Proteomics*.

[58] Baldwin MA (2004). Protein identification by mass spectrometry: issues to be considered. *Molecular and Cellular Proteomics*.

[59] Kinter M, Kinter CS *Application of Selected Reaction Monitoring to Highly Multiplexed Targeted Quantitative Proteomicsa Replacement for Western Blot Analysis*.

[60] Mahmood T, Yang PC (2012). Western blot: technique, theory, and trouble shooting. *North American Journal of Medical Sciences*.

[61] Lan N, Montelione GT, Gerstein M (2003). Ontologies for proteomics: towards a systematic definition of structure and function that scales to the genome level. *Current Opinion in Chemical Biology*.

[62] Smoot ME, Ono K, Ruscheinski J, Wang P-L, Ideker T (2011). Cytoscape 2.8: new features for data integration and network visualization. *Bioinformatics*.

[63] Senapati S, Barnhart K (2011). Managing endometriosis-associated infertility. *Clinical Obstetrics and Gynecology*.

[64] Cramer DW, Missmer SA (2002). The epidemiology of endometriosis. *Annals of the New York Academy of Sciences*.

[65] Missmer SA, Hankinson SE, Spiegelman D, Barbieri RL, Marshall LM, Hunter DJ (2004). Incidence of laparoscopically confirmed endometriosis by demographic, anthropometric, and lifestyle factors. *American Journal of Epidemiology*.

[66] Husby GK, Haugen RS, Moen MH (2003). Diagnostic delay in women with pain and endometriosis. *Acta Obstetricia et Gynecologica Scandinavica*.

[67] Gupta S, Goldberg JM, Aziz N, Goldberg E, Krajcir N, Agarwal A (2008). Pathogenic mechanisms in endometriosis-associated infertility. *Fertility and Sterility*.

[68] Buhimschi IA (2012). Using SELDI-TOF mass spectrometry on amniotic fluid and for clinical proteomics and theranostics in disorders of pregnancy. *Methods in Molecular Biology*.

[69] Defrère S, Lousse JC, González-Ramos R, Colette S, Donnez J, van Langendonckt A (2008). Potential involvement of iron in the pathogenesis of peritoneal endometriosis. *Molecular Human Reproduction*.

[70] Seo SK, Yang HI, Lee KE (2010). The roles of thioredoxin and thioredoxin-binding protein-2 in endometriosis. *Human Reproduction*.

[71] Pellicer A, Albert C, Mercader A, Bonilla-Musoles F, Remohí J, Simón C (1998). The follicular and endocrine environment in women with endometriosis: local and systemic cytokine production. *Fertility and Sterility*.

[72] McLaren J, Prentice A, Charnock-Jones DS, Smith SK (1996). Vascular endothelial growth factor (VEGF) concentrations are elevated in peritoneal fluid of women with endometriosis. *Human Reproduction*.

[73] Zhang H, Niu Y, Feng J, Guo H, Ye X, Cui H (2006). Use of proteomic analysis of endometriosis to identify different protein expression in patients with endometriosis versus normal controls. *Fertility and Sterility*.

[74] Seeber BE, Czech T, Buchner H (2010). The vitamin E-binding protein afamin is altered significantly in the peritoneal fluid of women with endometriosis. *Fertility and Sterility*.

[75] Zhou H, Ohno N, Terada N, Saitoh S, Fujii Y, Ohno S (2007). Involvement of follicular basement membrane and vascular endothelium in blood-follicle barrier formation of mice revealed by ‘*in vivo* cryotechnique’. *Reproduction*.

[76] Holmes DIR, Zachary I (2005). The vascular endothelial growth factor (VEGF) family: angiogenic factors in health and disease. *Genome Biology*.

[77] Ferrero S, Ragni N, Remorgida V (2006). Antiangiogenic therapies in endometriosis. *British Journal of Pharmacology*.

[78] Schäfer G, Cramer T, Suske G, Kemmner W, Wiedenmann B, Höcker M (2003). Oxidative stress regulates vascular endothelial growth factor-A gene transcription through Sp1- and Sp3-dependent activation of two proximal GC-rich promoter elements. *The Journal of Biological Chemistry*.

[79] Dennery PA (2007). Effects of oxidative stress on embryonic development. *Birth Defects Research C: Embryo Today: Reviews*.

[80] Slater M, Cooper M, Murphy CR (2007). The cytoskeletal proteins *α*-actinin, ezrin, and talin are de-expressed in endometriosis and endometrioid carcinoma compared with normal uterine epithelium. *Applied Immunohistochemistry and Molecular Morphology*.

[81] Harrington DJ, Lessey BA, Rai V (1999). Tenascin is differentially expressed in endometrium and endometriosis. *The Journal of Pathology*.

[82] Klemmt PAB, Carver JG, Koninckx P, McVeigh EJ, Mardon HJ (2007). Endometrial cells from women with endometriosis have increased adhesion and proliferative capacity in response to extracellular matrix components: towards a mechanistic model for endometriosis progression. *Human Reproduction*.

[83] Kijima T, Maulik G, Ma PC, Madhiwala P, Schaefer E, Salgia R (2003). Fibronectin enhances viability and alters cytoskeletal functions (with effects on the phosphatidylinositol 3-kinase pathway) in small cell lung cancer. *Journal of Cellular and Molecular Medicine*.

[84] Atkinson JC, Rühl M, Becker J, Ackermann R, Schuppan D (1996). Collagen VI regulates normal and transformed mesenchymal cell proliferation in vitro. *Experimental Cell Research*.

[85] Yang R, Nakamaki T, Lübbert M (1999). Cyclin A1 expression in leukemia and normal hematopoietic cells. *Blood*.

[86] Foyouzi N, Berkkanoglu M, Arici A, Kwintkiewicz J, Izquierdo D, Duleba AJ (2004). Effects of oxidants and antioxidants on proliferation of endometrial stromal cells. *Fertility and Sterility*.

[87] Li Z, Srivastava P (2004). APPENDIX 1T heat-shock proteins. *Current Protocols in Immunology*.

[88] Komatsu T, Konishi I, Fukumoto M (1997). Messenger ribonucleic acid expression of heat shock proteins HSP70 and HSP90 in human endometrium and myometrium during the menstrual cycle. *Journal of Clinical Endocrinology and Metabolism*.

[89] Matsuzaki S, Canis M, Vaurs-Barrière C (2004). DNA microarray analysis of gene expression profiles in deep endometriosis using laser capture microdissection. *Molecular Human Reproduction*.

[90] Kalmar B, Greensmith L (2009). Induction of heat shock proteins for protection against oxidative stress. *Advanced Drug Delivery Reviews*.

[91] Archer JS, Chang RJ (2004). Hirsutism and acne in polycystic ovary syndrome. *Best Practice & Research Clinical Obstetrics & Gynaecology*.

[92] The Rotterdam ESHRE/ASRM-Sponsored PCOS Consensus Workshop Group (2004). Revised 2003 consensus on diagnostic criteria and long-term health risks related to polycystic ovary syndrome. *Fertility and Sterility*.

[93] Wickenheisser JK, Nelson-DeGrave VL, McAllister JM (2006). Human ovarian theca cells in culture. *Trends in Endocrinology and Metabolism*.

[94] Gambineri A, Pelusi C, Vicennati V, Pagotto U, Pasquali R (2002). Obesity and the polycystic ovary syndrome. *International Journal of Obesity*.

[95] Escobar-Morreale HF, Luque-Ramírez M, San Millán JL (2005). The molecular-genetic basis of functional hyperandrogenism and the polycystic ovary syndrome. *Endocrine Reviews*.

[96] Insenser M, Montes-Nieto R, Murri M, Escobar-Morreale HF (2013). Proteomic and metabolomic approaches to the study of polycystic ovary syndrome. *Molecular and Cellular Endocrinology*.

[97] Rogalla T, Ehrnsperger M, Preville X (1999). Regulation of Hsp27 oligomerization, chaperone function, and protective activity against oxidative stress/tumor necrosis factor by phosphorylation. *The Journal of Biological Chemistry*.

[98] Kruszewski M (2003). Labile iron pool: the main determinant of cellular response to oxidative stress. *Mutation Research/Fundamental and Molecular Mechanisms of Mutagenesis*.

[99] Kawano Y, Narahara H, Miyamura K, Mifune K, Miyakawa I (1995). Inhibitory effect of transferrin on progesterone production in the granulosa cell of humans in vivo and porcine granulosa cell in vitro. *Gynecologic and Obstetric Investigation*.

[100] Oral O, Kutlu T, Aksoy E, Fiçicioğlu C, Uslu H, Tuğrul S (2006). The effects of oxidative stress on outcomes of assisted reproductive techniques. *Journal of Assisted Reproduction and Genetics*.

[101] Parthasarathy S, Santanam N, Ramachandran S, Meilhac O (2000). Invited review: potential role of oxidized lipids and lipoproteins in antioxidant defense. *Free Radical Research*.

[102] Gerke V, Moss SE (2002). Annexins: from structure to function. *Physiological Reviews*.

[103] Leon C, Nandan D, Lopez M, Moeenrezakhanlou A, Reiner NE (2006). Annexin V associates with the IFN-*γ* receptor and regulates IFN-*γ* signaling. *The Journal of Immunology*.

[104] Visse R, Nagase H (2003). Matrix metalloproteinases and tissue inhibitors of metalloproteinases: structure, function, and biochemistry. *Circulation Research*.

[105] Altindag O, Erel O, Aksoy N, Selek S, Celik H, Karaoglanoglu M (2007). Increased oxidative stress and its relation with collagen metabolism in knee osteoarthritis. *Rheumatology International*.

[106] Watanabe N, Ikeda U (2004). Matrix metalloproteinases and atherosclerosis. *Current Atherosclerosis Reports*.

[107] Smith S, Pfeifer SM, Collins JA (2003). Diagnosis and management of female infertility. *Journal of the American Medical Association*.

[108] Practice Committee of American Society for Reproductive Medicine (2012). Diagnostic evaluation of the infertile female: a committee opinion. *Fertility and Sterility*.

[109] Gleicher N, Barad D (2006). Unexplained infertility: does it really exist?. *Human Reproduction*.

[110] Heng S, Hannan NJ, Rombauts LJF, Salamonsen LA, Nie G (2011). PC6 levels in uterine lavage are closely associated with uterine receptivity and significantly lower in a subgroup of women with unexplained infertility. *Human Reproduction*.

[111] Cavagna M, Mantese JC (2003). Biomarkers of endometrial receptivity—a review. *Placenta*.

[112] Chen JI-C, Hannan NJ, Mak Y (2009). Proteomic characterization of midproliferative and midsecretory human endometrium. *Journal of Proteome Research*.

[113] Domínguez F, Garrido-Gómez T, López JA (2009). Proteomic analysis of the human receptive versus non-receptive endometrium using differential in-gel electrophoresis and MALDI-MS unveils stathmin 1 and annexin A2 as differentially regulated. *Human Reproduction*.

[114] González RR, Caballero-Campo P, Jasper M (2000). Leptin and leptin receptor are expressed in the human endometrium and endometrial leptin secretion is regulated by the human blastocyst. *Journal of Clinical Endocrinology and Metabolism*.

[115] Sakkas D, Lu C, Zulfikaroglu E, Neuber E, Taylor HS (2003). A soluble molecule secreted by human blastocysts modulates regulation of HOXA10 expression in an epithelial endometrial cell line. *Fertility and Sterility*.

[116] McReynolds S, Vanderlinden L, Stevens J, Hansen K, Schoolcraft WB, Katz-Jaffe MG (2011). Lipocalin-1: a potential marker for noninvasive aneuploidy screening. *Fertility and Sterility*.

[117] Jarkovska K, Martinkova J, Liskova L (2010). Proteome mining of human follicular fluid reveals a crucial role of complement cascade and key biological pathways in women undergoing in vitro fertilization. *Journal of Proteome Research*.

[118] Katz-Jaffe MG, Gardner DK, Schoolcraft WB (2006). Proteomic analysis of individual human embryos to identify novel biomarkers of development and viability. *Fertility and Sterility*.

[119] Katz-Jaffe MG, Schoolcraft WB, Gardner DK (2006). Analysis of protein expression (secretome) by human and mouse preimplantation embryos. *Fertility and Sterility*.

[120] Caplan JF, Filipenko NR, Fitzpatrick SL, Waisman DM (2004). Regulation of annexin A2 by reversible glutathionylation. *The Journal of Biological Chemistry*.

[121] Agarwal A, Said TM, Bedaiwy MA, Banerjee J, Alvarez JG (2006). Oxidative stress in an assisted reproductive techniques setting. *Fertility and Sterility*.

[122] Estes SJ, Ye B, Qiu W, Cramer D, Hornstein MD, Missmer SA (2009). A proteomic analysis of IVF follicular fluid in women ≤ 32 years old. *Fertility and Sterility*.

[123] Comeglio P, Fedi S, Liotta AA (1996). Blood clotting activation during normal pregnancy. *Thrombosis Research*.

[124] Afonso S, Romagnano L, Bablarz B (1997). The expression and function of cystatin C and cathepsin B and cathepsin L during mouse embryo implantation and placentation. *Development*.

[125] Nishio C, Yoshida K, Nishiyama K, Hatanaka H, Yamada M (2000). Involvement of cystatin C in oxidative stress-induced apoptosis of cultured rat CNS neurons. *Brain Research*.

[126] Wang HM, Zhang X, Qian D (2004). Effect of ubiquitin-proteasome pathway on mouse blastocyst implantation and expression of matrix metalloproteinases-2 and -9. *Biology of Reproduction*.

[127] Gomes-Marcondes MCC, Tisdale MJ (2002). Induction of protein catabolism and the ubiquitin-proteasome pathway by mild oxidative stress. *Cancer Letters*.

